# Assessing the excess costs of the in-hospital adverse events covered by the AHRQ’s Patient Safety Indicators in Switzerland

**DOI:** 10.1371/journal.pone.0285285

**Published:** 2024-02-05

**Authors:** Alice Giese, Rasheda Khanam, Son Nghiem, Anthony Staines, Thomas Rosemann, Stefan Boes, Michael M. Havranek

**Affiliations:** 1 Competence Center for Health Data Science, Faculty of Health Sciences and Medicine, University of Lucerne, Lucerne, Switzerland; 2 School of Business and Centre for Health Research, University of Southern Queensland, Toowoomba, Queensland, Australia; 3 Institute of Primary Care, University of Zurich and University Hospital Zurich, Zurich, Switzerland; 4 College of Health & Medicine, Australian National University, Canberra, Australian Capital Territory, Australia; 5 IFROSS Institute, University of Lyon III, Lyon, France; 6 Hospital Federation of Vaud, Prilly, Vaud, Switzerland; Taipei Medical University, TAIWAN

## Abstract

There currently exists no comprehensive and up-to date overview on the financial impact of the different adverse events covered by the Patient Safety Indicators (PSIs) from the Agency for Healthcare Research and Quality. We conducted a retrospective case-control study using propensity score matching on a national administrative data set of 1 million inpatients in Switzerland to compare excess costs associated with 16 different adverse events both individually and on a nationally aggregated level. After matching 8,986 cases with adverse events across the investigated PSIs to 26,931 controls, we used regression analyses to determine the excess costs associated with the adverse events and to control for other cost-related influences. The average excess costs associated with the PSI-related adverse events ranged from CHF 1,211 (PSI 18, obstetric trauma with instrument) to CHF 137,967 (PSI 10, postoperative acute kidney injuries) with an average of CHF 27,409 across all PSIs. In addition, adverse events were associated with 7.8-day longer stays, 2.5 times more early readmissions (within 18 days), and 4.1 times higher mortality rates on average. At a national level, the PSIs were associated with CHF 347 million higher inpatient costs in 2019, which corresponds to about 2.2% of the annual inpatient costs in Switzerland. By comparing the excess costs of different PSIs on a nationally aggregated level, we offer a financial perspective on the implications of in-hospital adverse events and provide recommendations for policymakers regarding specific investments in patient safety to reduce costs and suffering.

## Introduction

In-hospital adverse events cause suffering and harm and result in longer hospitalization stays and more frequent readmissions and deaths [1]. One in ten patients (range 2.9–21.9%) suffers from at least one adverse event during hospitalization [2]. These are international figures, but the situation in Switzerland is no different [3]. Approximately half of these adverse events are considered potentially preventable [2, 4–6]. In addition to causing grief, these adverse events increase costs in the healthcare system [7]. A conservative assumption from the 2017 Organisation for Economic Co-operation and Development (OECD) report [8], as well as a Canadian study [9], suggests that between 1.3% and 32% of public hospital spending is related to the treatment of adverse events. In the US, adverse events caused excess costs of approximately USD 17.1 billion in 2008, which was 0.72% of the USD 2.39 trillion spent on healthcare during that year [10].

Most previous studies on the costs of in-hospital adverse events only focused on some selected adverse events such as medical errors (see, e.g., [7, 11]). However, to allow for comparisons of the economic consequences of various adverse events, a set of different adverse events must be measured simultaneously according to exact and agreed-upon specifications. The Agency for Healthcare Research and Quality (AHRQ) utilizes an evidence-based methodology for identifying adverse events associated with patient safety using hospital administrative data. Initially, 19 Patient Safety Indicators (PSIs) were proposed, and these were then gradually reduced to 17 (version v2021). The remaining PSIs offer a standardized and well-structured set of indicators to assess hospitals patient safety that is used, for example, for provider comparisons in the US and country comparisons across the OECD.

Although the PSIs were introduced nearly 20 years ago, only a few studies have analyzed their financial impact. Furthermore, most researchers who have estimated the excess costs associated with PSI-related adverse events either used individualized definitions or only examined a select subset of PSIs. For example, Rivard, Luther, Christiansen, Zhao, Loveland, Elixhauser, et al. [12], calculated costs for only nine PSIs, and Encinosa and Hellinger [13] categorized the PSIs into seven groups, which prevented such studies from analyzing the financial impact across all individual PSIs. The most comprehensive study to date was conducted by Zhan and Miller [14] in 2003. They demonstrated that patients experiencing one of these adverse events were hospitalized up to 10.89 days longer, had excess costs of up to USD 57,727, and excess mortality of up to 21.96%. However, even Zhan and Miller [14] did not include all PSIs in their analysis. Furthermore, they did not take the frequency of the PSIs into account when evaluating the financial impact of the different PSIs on overall national inpatient costs.

We aimed to close these research gaps and show the excess costs associated with each adverse event across all individual PSIs and how they contribute to the total national excess costs of PSI-related adverse events. Thus, by comparing the PSIs in terms of cost and frequency, we can draw conclusions about the national financial impact of the different PSI-related adverse events and inform policy decisions regarding patient safety priorities from an economic perspective.

## Methods

### Data

This retrospective cohort study was conducted in Switzerland using two national health administration and finance data sets from 2019 provided by the Swiss Federal Statistical Office. The first data set [15] contained all inpatient cases treated in Swiss hospitals during 2019 with their diagnosis codes (ICD-10-GM) [16], procedure codes (CHOP) [17], diagnosis-related group (Swiss-DRG) [18], and other clinically relevant variables, such as the admission and discharge conditions, as well as demographic information like age and sex. However, this data set did not include financial information on the cases. The second data set included additional case-related cost data obtained from the cost-unit accounts consisting of a “bottom-up” cost analysis where all the direct and indirect costs (i.e., the resources spent) were attributed to the hospital stays [19]. However, this second data set was restricted to acute inpatient cases (excluding psychiatric and rehabilitation cases, which are reimbursed outside of the DRG system in Switzerland) and contained only the 84% of patients who had no private supplementary insurance coverage. This combined (second) data set (administrative health data and cost data per case) was used for our cost analyses. It included 1,012,270 cases in total, of which 331,301 (32.7%) were excluded either because they did not meet the inclusion criteria of one of the PSI samples (e.g., the patients that were under 18 years old) or because the patients did not receive any treatment codes as procedure during the hospitalization (7.6%). The latter exclusion criterion was applied because of the presence of so-called “nursing- or waiting patients” in the Swiss administrative data (i.e., cases that naturally have much lower resource requirements) to prevent comparisons of cases with PSI-related adverse events to cases without any treatment. After these exclusions, 680,969 cases remained and were used in our analyses (see also Table 2, which gives the exact sample sizes of each PSI). In contrast, the other data set (which also included patients with private supplementary insurance coverage but did not contain any financial information) was used to determine the total number of PSI-related adverse events in 2019 to estimate the national financial impact of the PSIs (see Section 2.3.3 below).

### PSI samples and the dependent variable

Adverse events were assessed using the PSI definitions utilized by the AHRQ [20], translated into the Swiss medical coding systems (ICD-10-GM and CHOP). The initial translation of the PSI specifications was performed as part of a project funded by the Swiss Innovation Agency (Innosuisse), which sought to make several international quality indicator sets available in Switzerland. It was subsequently checked by two independent medical coding experts and validated in a large record review study with nine Swiss hospital partners (publication in submission). Of the 17 current hospital-level indicators that screen for hospitalizations with potentially preventable adverse events, PSI 15 (accidental abdominopelvic punctures or lacerations) had to be excluded from our cost analyses because of its rarity (*n* = 15). The remaining 16 PSIs were included in our analyses (see Table 1).

**Table 1:** Patient Safety Indicators (PSIs).

PSI	AHRQ description	Short form used in the main text
PSI 02	Death in low-mortality diagnosis-related groups	Death in low-mortality DRGs
PSI 03	Pressure ulcer	Pressure ulcer
PSI 04	Death among surgical inpatients with serious treatable complications	Death after serious complications
PSI 05	Retained surgical item or unretrieved device fragment count	Retained surgical items
PSI 06	Iatrogenic pneumothorax	Iatrogenic pneumothorax
PSI 07	Central venous catheter-related bloodstream infection	CVC bloodstream infection
PSI 08	In-hospital fall with hip fracture	Fall with hip fracture
PSI 09	Postoperative hemorrhage or hematoma	Postoperative hemorrhage/hematoma
PSI 10	Postoperative acute kidney injury requiring dialysis	Postoperative acute kidney injury
PSI 11	Postoperative respiratory failure	Postoperative respiratory failure
PSI 12	Perioperative pulmonary embolism or deep vein thrombosis	Perioperative embolism or thrombosis
PSI 13	Postoperative sepsis	Postoperative sepsis
PSI 14	Postoperative wound dehiscence	Wound dehiscence
PSI 15	Unrecognized abdominopelvic accidental puncture or laceration (excluded from analyses, see main text)	Accidental punctures or lacerations
PSI 17	Birth trauma–injury to neonate	Birth trauma
PSI 18	Obstetric trauma–vaginal delivery with instrument	Obstetric trauma with instrument
PSI 19	Obstetric trauma–vaginal delivery without instrument	Obstetric trauma without instrument

*Note*. PSI 15 had an insufficient number of cases to be used for analysis but is depicted here as well for the sake of completeness. Short form: Subsequently, we will use these abbreviations to refer to the PSIs in the main text.

It must be noted that in Switzerland, the encounter-level cost data from hospitals is available upon request for research studies. Thus, in contrast to previous studies from other countries examining charges, the dependent variable we focused on was the total cost per case obtained from the bottom-up full-cost method that allocates every type of cost (even overheads) to the treated case [21]. This approach is considered appropriate for measuring excess costs due to adverse events [22]. Costs are subsequently provided in Swiss francs (CHF), which can be converted into US dollars (USD) with an exchange rate (June 2022) of 0.99 (USD 1 = CHF 0.99) and into euros (EUR) with an exchange rate of 1.04 (EUR 1 = CHF 1.04). In addition to our primary outcome variable costs, we also investigated several secondary outcome variables, such as length of stay, mortality, and number of readmissions.

### Statistical analysis

After building the PSI samples and identifying the PSI-related adverse events (based on the PSI definitions of the AHRQ) [20], we examined population characteristics, such as the number of patients per PSI sample and the PSIs’ incidences. Please note that the subsequently reported incidence rates are always considered in relation to the corresponding PSI sample (rather than in comparison to the entire data set).

#### Matching

Similar to previous studies investigating the costs of adverse events [12–14, 23, 24], we used propensity score matching to compare PSI cases with matched control cases possessing similar characteristics. The selection of the control cases was restricted to the patient populations defined by the different PSI specifications. Caution was needed to select only control variables for the matching that predicted the occurrence of a PSI-related adverse event but that were not caused by the adverse events covered by the PSIs [25]. For example, based on the findings of preliminary analyses, we did not include DRG codes as matching variables because, according to the grouper methodology used by the Swiss DRG system, the presence of PSI-related complications determines which DRGs patients fall in.

Propensity score matching was performed separately for each PSI. Based on recommendations from Austin [26], we matched the samples on the logit of the propensity score using calipers of width equal to 0.2 of the standard deviation of the logit of the propensity score based on the following variables:

- age (using five-year age groups);
- sex;
- nationality (Swiss or foreign);
- admission as an emergency (admission type = emergency OR admitted by = ambulance);
- admission from a nursing home;
- transfer from another hospital; and
- the Elixhauser Index, which was recently validated and recalculated on Swiss data [27].

Apart from age and the Elixhauser Index, all matching variables were coded in binary format. In addition, some of the used matching variables had to be excluded in certain PSIs. For example, the variables “sex” and “admission from a nursing home” were not relevant for obstetrical PSIs like PSI 18 (obstetric trauma with instrument) and PSI 19 (obstetric trauma without instrument). On the other hand, for PSI 17 (birth trauma), we had to include additional matching variables that were relevant to the circumstances of the newborns. These included gestational age (in days), birth weight (in grams), head size (in centimeters), and the mother’s number of previous live births [28].

We report the area under the receiver operating characteristic curve (AUC) to examine the accuracy of our propensity score models in predicting PSI cases. To ensure that the matched samples would have sufficient statistical power even though PSI-related adverse events are rare, we used a nearest neighbor 1:3 matching (instead of the more traditional 1:1 matching) [29]. However, since 1:1 matching has been used more frequently in previous studies, we also provide results of a 1:1 matching in the supplementary material (S1–S4 Tables in S2 Appendix) to demonstrate similar findings under different matching conditions.

#### Excess costs per PSI

After matching, we assessed the potentially remaining differences between PSI cases and matched controls using the Mann-Whitney U and chi-squared tests. Next, we performed ordinary least squares (linear) regression analyses for each PSI sample separately as well as for all PSI cases and controls combined to investigate the association of the PSI occurrence with costs. A combined regression was estimated without distinguishing between the PSI-related adverse events to examine the association of average excess costs with the adverse events across all PSIs. Given that certain residual differences in matching variables remained between PSI cases and matched controls, we followed the advice of Austin [26] to also include the matching variables as covariates in our regression analyses in order to reduce potentially remaining biases.

In addition, and analogous to the approach taken by Marbus, Schweitzer, Groeneveld, Oosterheert, Schneeberger, van der Hoek, et al. [30], we controlled for the premature death of patients in our analyses. Dying “prematurely” was defined as deaths occurring with a length of stay below the lower-trim point set out in the respective DRG of the patient. In this manner, we allow for comparisons of the excess costs of different adverse events independent of the potential effect of more frequent premature deaths in certain PSIs. This was necessary because some PSIs were previously shown [14] to have higher mortality rates than others, which was hypothesized to potentially and artificially reduce costs if patients die prematurely and no longer receive the otherwise required treatments.

In summary, we used the following regression model to explain the total costs of patients denoted by *y*_*i*_ using our set of explanatory variables denoted by *x*_*k*,*I*_:

$$y_{i}=\beta_{0}+\beta_{1}x_{1,i}+\beta_{2}x_{2,i}+\beta_{3}x_{3,i}+\beta_{4}x_{4,i}+\beta_{5}x_{5,i}+\beta_{6}x_{6,i}+\beta_{7}x_{7,i}+\beta_{8}x_{8,i}+\beta_{9}x_{9,i}+\varepsilon i$$ (1)

for *i* = 1, …, *N*_pat_, where

- *x*_*1*,*i*_ is the occurrence of the respective PSI (binary);
- *x*_*2*,*i*_ *… x*_*8*,*i*_ are the included covariates (age, sex, nationality, admission as emergency; admission from a nursing home, transfer from another hospital, and the Elixhauser Index);
- *x*_*9*,*i*_ is premature death during hospitalization (binary); and
- *ε*_*i*_ is the error term for patient *i* with heteroscedastic variance *σ*^2^_*HC*,*i*_.

#### Excess costs at the national level

To obtain an accurate cost estimate of the financial impact associated with PSI-related adverse events at a national level, excess costs associated with adverse events covered by the PSIs were multiplied by their frequency. However, because in preliminary analyses we noticed that certain PSIs, such as PSI 10 (postoperative acute kidney injury), PSI 11 (postoperative respiratory failure), and PSI 13 (postoperative sepsis), often co-occurred, we computed an additional regression analysis for all PSI cases and their matched controls combined. In contrast to the combined regression introduced above, we also investigated the individual effect of each PSI-related adverse event by including a separate variable for each PSI in the regression model. This procedure allowed us to control for the co-occurrence of certain PSIs and avoided double-counting co-occurring PSIs in our national aggregate. Furthermore, we did not control for the premature death of patients in this regression analysis, since our goal was to determine the most accurate estimate of the actual financial impact and adjusting the costs for prematurely deceased patients would have overestimated the national costs.

Next, the regression coefficient of each PSI (i.e., the excess costs that this adverse event generated on average per case) was multiplied by the frequency of the adverse event covered by that PSI. This frequency was, assessed on the full national data set (as was explained in Section 2.1 above). It must be noted, however, that this estimate only includes patients who met the inclusion criteria of the PSI samples, whereas certain high-risk patient groups are specifically excluded in the sample specifications of the PSIs (for further information, see the discussion in Section 4).

The only exceptions from this procedure for calculating national costs were the obstetrical PSIs 17–19. These PSIs and their samples were not included in the combined regression, since obstetrical cases require much lower resources (i.e., have lower costs) than other cases. This prevented us from computing the associated excess costs of the adverse events covered by PSIs 17–19 alongside the other PSIs. Their coefficients were, therefore, obtained from their individual regressions. We consider this to be correct, as these PSIs did not co-occur with others nor did any of the patients die. For all statistical analyses, the software IBM SPSS Statistics 27 was used, and results were considered significant where *p* < .05.

## Results

### Study population and matching

During the observed period, 9,853 *different* PSI-related adverse events were registered among the 680,969 inpatients investigated, and 9,109 patients experienced *at least one* adverse event (see Table 2). PSI 09 (postoperative hemorrhage/hematoma) was the most common event, accounting for 27.6% of the adverse events across all PSIs, followed by PSI 12 (perioperative embolism or thrombosis), which accounted for 14.1% of the adverse events among the PSIs. The rarest PSI among those considered was PSI 02 (death in low-mortality DRGs), which, with 43 cases, accounted for only 0.4% of the measured patient safety-adverse events.

**Table 2:** Sample characteristics and matching results.

		Raw data		Incidence	After matching		Matching rate	AUC
		*N* (PSI = 0)	*N* (PSI = 1)	Per thousand	*N* (PSI = 0)	*N* (PSI = 1)		
PSI 02	Death in low-mortality DRGs	210,113	43	0.20	129	43	100.0%	0.919
PSI 03	Pressure ulcer	207,566	865	4.15	2,595	865	100.0%	0.744
PSI 04	Death after serious complications	6,511	1,333	169.94	1,555	524	39.3%	0.731
PSI 05	Retained surgical items	666,990	76	0.11	228	76	100.0%	0.612
PSI 06	Iatrogenic pneumothorax	557,683	310	0.56	930	310	100.0%	0.726
PSI 07	CVC bloodstream infection	467,237	553	1.18	1,659	553	100.0%	0.826
PSI 08	Fall with hip fracture	186,552	111	0.59	333	111	100.0%	0.925
PSI 09	Postoperative hemorrhage/hematoma	303,722	2,717	8.87	8,151	2,717	100.0%	0.634
PSI 10	Postoperative acute kidney injury	230,484	227	0.98	644	217	95.6%	0.956
PSI 11	Postoperative respiratory failure	197,181	90	0.46	264	88	97.8%	0.921
PSI 12	Perioperative embolism or thrombosis	316,278	1,393	4.39	4,165	1,389	99.7%	0.861
PSI 13	Postoperative sepsis	231,525	493	2.12	1,421	474	96.1%	0.906
PSI 14	Wound dehiscence	43,545	151	3.46	453	151	100.0%	0.792
PSI 15	Accidental punctures or lacerations	43,794	15	-	-	-	-	-
PSI 17	Birth trauma	12,865	101	7.79	234	78	77.2%	0.706
PSI 18	Obstetric trauma with instrument	7,515	560	69.35	1,680	560	100.0%	0.530
PSI 19	Obstetric trauma without instrument	39,964	830	20.35	2,490	830	100.0%	0.546

*Note*. *AUC* area under the curve. The matching rate indicates the proportion of cases that met the common support assumption. PSI 15 had an insufficient number of cases to be used for analysis but is depicted here as well for the sake of completeness.

We matched 8,986 PSI cases to 26,931 control cases based on the matching variables introduced in Section 2.3.1. The predicted probabilities from our propensity score models showed good to excellent AUC values across most PSIs (see Table 2, [31]) except for PSI 18 (obstetric trauma with instrument) and PSI 19 (obstetric trauma without instrument), for which we lacked obstetrically-relevant predictor variables. Thanks to the high number of cases available, a matching rate > 95% was achieved for almost all PSIs. Lower matching rates were only registered for PSI 04 (death after serious complications), which focuses on a severely diseased patient sample for which it was difficult to find controls that did not die and, to a lesser degree, PSI 17 (birth trauma).

The comparisons of means and relative frequencies in the variables used for the matching are provided in the supplement (S1.1 Table in S1 Appendix). Rare residual differences in specific variables (mainly age and the Elixhauser Index) still existed after matching, which is why we subsequently included all matching variables as covariates in the regression models (see also Section 2.3.2). However, most comparisons of the matching variables (93%) showed no significant differences between the matched groups.

Table 3 compares the means and relative frequencies of the outcome variables between the matched groups. It shows that PSI cases are associated with significantly higher costs (with a weighted mean of CHF 56,439 across all PSI cases) than their matched controls (with a weighted mean of CHF 23,696 across all matched non-PSI cases), resulting in a weighted mean difference of CHF 32,743. Put differently, adverse events are associated with 15% excess costs in PSI 18 (obstetric trauma with instrument) and 335% excess costs in PSI 10 (postoperative acute kidney injury).

**Table 3:** Comparisons of means and relative frequencies across outcome variables in the matched groups.

PSI		Total cost in CHF		LOS		No. of early readmissions		Mortality	
		0	1	0	1	0	1	0	1
PSI 02: Death in low-mortality DRGs	Mean/RF	9,432.97	16,211.84	5.05	5.81	0.03	0.02	0.00%	100.00%
	SD	7,617.84	18,100.84	5.19	6.57	0.17	0.15	-	-
	test statistic	-1.61		-0.11		-0.26		172.00	
	p	.107		.911		.794		< .001 ***	
PSI 03: Pressure ulcer	Mean/RF	22,261.30	51,792.02	12.65	23.62	0.07	0.07	5.66%	16.99%
	SD	31,696.64	77,644.00	10.13	24.21	0.27	0.26	-	-
	test statistic	-18.05		-17.95		-0.80		107.10	
	p	< .001 ***		< .001 ***		.426		< .001 ***	
PSI 04: Death after serious compli-cations	Mean/RF	85,827.49	101,459.06	25.96	16.87	0.10	0.05	0.00%	100.00%
	SD	115,484.61	128,691.57	23.62	20.07	0.31	0.23	-	-
	test statistic	-3.08		-12.40		-3.76		2079.00	
	p	.002 **		< .001 ***		< .001 ***		< .001 ***	
PSI 05: Retained surgical items	Mean/RF	13,932.12	30,748.18	5.51	10.78	0.03	0.07	2.19%	2.63%
	SD	20,026.31	47,458.73	6.65	18.54	0.19	0.25	-	-
	test statistic	-4.67		-4.01		-1.84		0.05	
	p	< .001 ***		< .001 ***		.066		>.999	
PSI 06: Iatrogenic pneumo-thorax	Mean/RF	17,998.51	43,259.37	8.43	12.71	0.05	0.05	4.62%	9.35%
	SD	31,285.92	59,343.67	11.05	12.86	0.23	0.21	-	-
	test statistic	-11.93		-6.72		-0.60		9.52	
	p	< .001 ***		< .001 ***		.551		.002 **	
PSI 07: CVC bloodstream infection	Mean/RF	25,063.80	96,456.09	10.42	28.25	0.07	0.11	6.21%	10.85%
	SD	43,110.11	120,722.84	11.05	24.59	0.27	0.32	-	-
	test statistic	-23.68		-22.64		-3.78		13.09	
	p	< .001 ***		< .001 ***		< .001 ***		< .001 ***	
PSI 08: Fall with hip fracture	Mean/RF	35,075.09	65,256.16	11.32	24.60	0.08	0.06	9.01%	17.12%
	SD	46,848.67	54,699.80	13.85	20.12	0.28	0.24	-	-
	test statistic	-7.33		-8.05		-0.53		5.57	
	p	< .001 ***		< .001 ***		.597		.018 *	
PSI 09: Post-operative hemorrhage/hematoma	Mean/RF	18,145.03	35,766.92	6.20	11.84	0.04	0.25	1.36%	2.50%
	SD	29,283.71	48,453.42	9.20	14.41	0.19	0.48	-	-
	test statistic	-31.45		-30.09		-31.42		16.38	
	p	< .001 ***		< .001 ***		< .001 ***		< .001 ***	
PSI 10: Post-operative acute kidney injury	Mean/RF	41,325.22	179,900.37	12.38	32.29	0.05	0.06	3.57%	36.87%
	SD	49,047.81	183,709.60	13.60	31.43	0.22	0.23	-	-
	test statistic	-17.00		-11.83		-0.41		170.84	
	p	< .001 ***		< .001 ***		.679		< .001 ***	
PSI 11: Post-operative respiratory failure	Mean/RF	35,207.06	140,475.73	10.56	31.38	0.03	0.06	5.30%	23.86%
	SD	53,024.94	83,730.84	13.08	18.67	0.16	0.28	-	-
	test statistic	-11.36		-10.60		-0.90		25.39	
	p	< .001 ***		< .001 ***		.370		< .001 ***	
PSI 12: Peri-operative embolism or thrombosis	Mean/RF	36,195.73	71,827.91	11.81	21.62	0.06	0.10	6.31%	10.15%
	SD	60,247.28	112,642.46	16.01	22.60	0.26	0.31	-	-
	test statistic	-23.64		-22.91		-4.57		22.73	
	p	< .001 ***		< .001 ***		< .001 ***		< .001 ***	
PSI 13: Post-operative sepsis	Mean/RF	31,497.43	124,930.27	9.90	32.14	0.04	0.11	2.39%	22.36%
	SD	41,041.90	144,215.25	12.37	27.60	0.21	0.31	-	-
	test statistic	-24.12		-23.45		-5.40		207.18	
	p	< .001 ***		< .001 ***		< .001 ***		< .001 ***	
PSI 14: Wound dehiscence	Mean/RF	33,965.91	78,382.95	12.52	26.76	0.06	0.17	5.08%	10.60%
	SD	53,418.18	63,757.46	16.75	14.48	0.25	0.40	-	-
	test statistic	-13.55		-12.80		-3.90		5.71	
	p	< .001 ***		< .001 ***		< .001 ***		.017 *	
PSI 17: Birth trauma	Mean/RF	5,852.45	12,615.03	4.82	6.36	0.01	0.03	-	-
	SD	14,061.31	20,300.98	4.89	5.97	0.11	0.16	-	-
	test statistic	-5.38		-2.16		-0.78		-	
	p	< .001 ***		.030 *		.436		-	
PSI 18: Obstetric trauma with instrument	Mean/RF	8,195.47	9,400.23	4.06	4.30	0.00	0.00	-	-
	SD	3,514.32	3,445.03	1.55	1.43	0.00	0.00	-	-
	test statistic	-8.80		-4.08		0.00		-	
	p	< .001 ***		< .001 ***		-		-	
PSI 19: Obstetric trauma without instrument	Mean/RF	6,415.47	7,851.62	3.47	3.83	0.00	0.00	-	-
	SD	2,450.17	2,689.39	1.23	1.18	0.02	0.00	-	-
	test statistic	-14.83		-8.45		-0.58		-	
	p	< .001 ***		< .001 ***		.564		-	

*Note*.

*** = *p* < .001;

** = *p* < 0.01;

* = *p* < 0.05;

No. = number; Binary variables are expressed as relative frequencies (RF) in percent (%). Continuous variables are expressed as means and standard deviations (SD). *CHF* Swiss francs, *LOS* length of stay, *p* probability value. Please note that two separate cases of the same patient are reported as one single case in the administrative data set we used if the second case is an early hospital readmission within 18 days after discharge in the same major diagnostic category (MDC). This is the reason why cases flagged as deaths within PSI 02 and PSI 04 can have an intermediate discharge and readmission.

In addition to these costs, the average length of stay was associated with a 1.9 times higher, and therefore 7.8 days longer, length of stay in PSI-related adverse event cases compared to controls. In particular, patients with PSI 13 (postoperative sepsis) stayed 22.3 days longer, while patients with PSI 18 (obstetric trauma with instrument) or PSI 19 (obstetric trauma without instrument) only stayed an extra several hours (see Table 3). Additionally, the PSI-related adverse event cases were associated with 2.5 times higher early readmission rates (within 18 days, 12.0%) than their matched controls (4.8%). While, for example, patients with PSI 09 (postoperative hemorrhage/hematoma) had a seven times higher early readmission rate. Furthermore, the mortality rate was 4.1 times higher in cases with adverse events (16.7%) compared to their controls (4.1%). While patients with PSI 10 (postoperative acute kidney injury) had a 10.3 times higher mortality rate (36.9% instead of 3.6%) and patients with PSI 13 (postoperative sepsis) had a 9.4 times higher mortality rate (22.4% instead of 2.4%), in the obstetrical PSIs (PSI 17–19) no patients died. If deaths from PSI 02 (death in low-mortality DRG) and PSI 04 (death after serious complications) are excluded, the mortality rate is still 2.2 times higher (9.2%) in cases with adverse events compared to their controls.

### Excess costs across individual PSIs

We used regression analyses to examine the association of the PSI-related adverse events with the total costs per case independent of several covariates. The individual regression analyses per PSI and an overview of the individual regression analyses is provided in the supplemental material (S2–S18 Tables in S1 Appendix). In contrast, Table 4 presents the results of a regression analysis across all PSIs and matched controls (without distinguishing between PSIs) to enable a discussion of the average excess costs across all PSIs.

**Table 4:** Regression analysis using the occurrence of all the PSIs (without distinguishing the kind of PSI) and several covariates to explain total costs (in CHF).

Constant	B (in CHF)	Std. Error	t	p	
	7,364.61	990.39	7.436	< .001	***
PSI occurrence	27,408.85	664.91	41.222	< .001	***
Age	-65.59	15.98	-4.105	< .001	***
Sex: male	7,008.03	626.82	11.180	< .001	***
Nationality: Swiss	-1,581.37	715.02	-2.212	.027	*
Admission: Emergency	5,084.45	606.34	8.385	< .001	***
Admission: From nursing home	-3,667.48	2,478.05	-1.480	.139	
Admission: Transferred	25,242.13	1,226.06	20.588	< .001	***
Elixhauser Index	1,267.29	23.06	54.946	< .001	***
Premature death	-27,692.78	3,479.83	-7.958	< .001	***

*Note*.

*** = *p* < .001;

** = *p* < 0.01;

* = *p* < 0.05;

*B* unstandardized beta coefficients (costs), *t* test statistic, *p* probability value, *CHF* Swiss francs.

**Fig 1:**
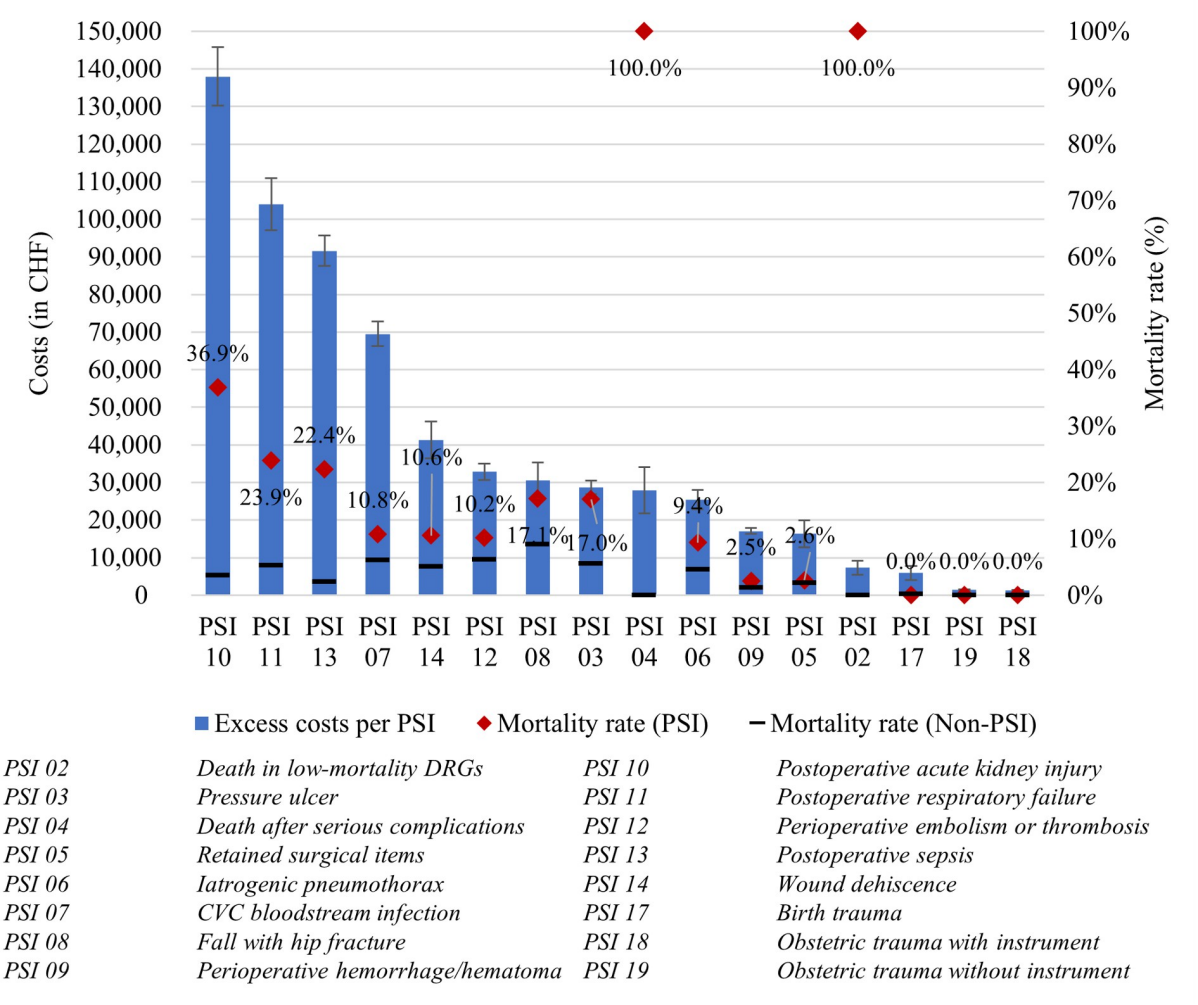
Excess costs and mortality rates associated with the different PSIs (according to their individual regression models, see S2-S17 Tables in S1 Appendix). *Note*. Error bars illustrate standard deviations. Mortality rates are shown as averages in PSI samples vs. in the control group (non-PSI). PSI 2 (death in low-mortality DRGs) and PSI 4 (death after serious complications) have a 100% mortality rate since these PSIs assess death as outcome of interest. None of the patients in the samples of PSI 17, PSI 18, and PSI 19 died.

Fig 1 illustrates the excess costs associated with each PSI in descending order of magnitude (see also S18 Table in S1 Appendix) together with their mortality rates. As can be seen, excess costs vary across PSIs, and the average excess costs associated with all PSIs are CHF 27,409 (see Table 4). PSI 10 (postoperative acute kidney injuries), PSI 11 (postoperative respiratory failure), PSI 13 (postoperative sepsis), and PSI 07 (CVC bloodstream infection) were associated with higher costs than the other PSIs. Apart from PSI 02 (death in low-mortality DRGs) and PSI 04 (death after serious complications), these are also the PSIs with the highest mortality rates. On the other end of the spectrum, the obstetrical PSI 17 (birth trauma), PSI 18 (obstetric trauma with instrument), and PSI 19 (obstetric trauma without instrument), together with PSI 02 (death in low-mortality DRGs) and PSI 05 (retained surgical items), are associated with the lowest excess costs across all PSIs.

The number of premature deaths across the PSIs are provided in the supplemental material (S1.2 Table in S1 Appendix). It shows that the number of premature deaths is low across most PSIs. The individual regression results provided in the supplemental material (S2–S17 Tables in S1 Appendix) also show that premature death is associated with reduced costs in PSI 02 (death in low-mortality DRGs), PSI 04 (death after serious complications), PSI 06 (iatrogenic pneumothorax), PSI 07 (CVC bloodstream infection), PSI 10 (postoperative acute kidney injury), PSI 12 (perioperative embolism or thrombosis), and PSI 13 (postoperative sepsis). In the combined sample (see Table 4), associated costs were, on average, CHF 27,693 (*p* = < .000) lower across all PSIs in cases of premature death. However, as S1.2 Table in S1 Appendix illustrates, these cases of premature death are relatively rare across most PSIs.

### Excess costs at a national level

**Fig 2:**
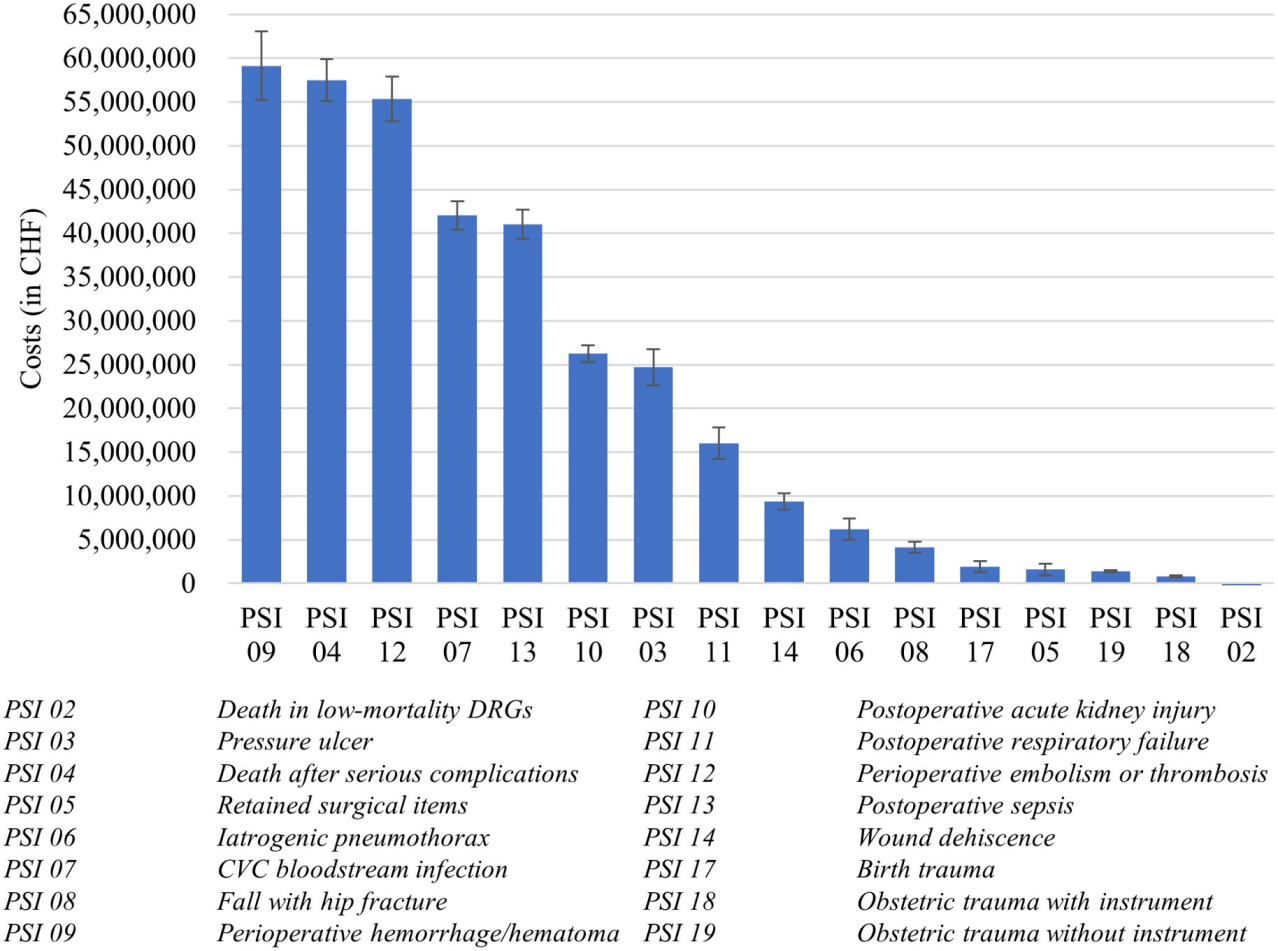
Total national costs associated with each PSI incurred in Switzerland in the fiscal year of 2019. *Note*. Error bars illustrate standard deviations. The calculations that underlie these results can be found in the S18 and S19 Tables in S1 Appendix.

Fig 2 demonstrates the aggregated national costs associated with each PSI in Switzerland in 2019. These costs were calculated by multiplying the excess costs associated with each PSI by the number of adverse events per PSI (see S18, S19 Tables in S1 Appendix and Section 2.3.3). Comparing Figs 1 and 2 reveals that, on a nationally aggregated level, PSI 07 (CVC bloodstream infection) and PSI 13 (postoperative sepsis) are still associated with comparably high excess costs after taking their frequency into account. However, in addition, PSI 09 (postoperative hemorrhage/hematoma), PSI 04 (death after serious complications), and PSI 12 (perioperative embolism or thrombosis) are now also among the most costly adverse events when their high frequency is considered. In contrast, we still find the same PSIs as in Fig 1 to be among the least costly, namely PSI 17 (birth trauma), PSI 18 (obstetric trauma with instrument), and PSI 19 (obstetric trauma without instrument), as well as PSI 02 (death in low-mortality DRGs) and PSI 05 (retained surgical items).

Finally, by summing up the total excess costs associated with all patient safety-adverse events (see S19 Table in S1 Appendix), the total national excess costs associated with PSI-related adverse events in Switzerland in 2019 were estimated at CHF 347 million.

## Discussion

There are many reasons to increase patient safety and reduce in-hospital adverse events. However, instead of focusing on ethical, regulatory, or patient-centered reasons, we investigated the financial impact of patient safety-adverse events in order to motivate improvements. Across all PSIs combined, the occurrence of a PSI-related adverse event was associated with an average excess cost of CHF 27,409. Among the specific PSI samples, PSI 10 (postoperative acute kidney injury) was associated with the highest excess costs per case, followed by PSI 11 (postoperative respiratory failure) and PSI 13 (postoperative sepsis). However, if the volume of the PSIs is taken into account, the highest costs were associated with PSI 09 (postoperative hemorrhage/hematoma), followed by PSI 04 (death after serious complications) and PSI 12 (perioperative embolism or thrombosis).

### Comparing excess costs and incidences of PSIs with previous studies

Our analyses confirmed that both per-event excess costs as well as the volume of PSIs matter when assessing the impact of in-hospital adverse events on the health system., Comparing these findings to the selection of PSIs that the OECD annually assesses, benchmarks, and publishes for European countries reveals that its PSI selection does not match the economic relevance of the PSIs (at least for Switzerland). Although two of the PSIs it reports, namely PSI 12 (perioperative embolism or thrombosis) and PSI 13 (postoperative sepsis), have shown an important financial impact in our study. The other four PSIs in the OECD’s reports, PSI 05 (retained surgical items), PSI 14 (wound dehiscence), PSI 18 (obstetric trauma with instrument), and PSI 19 (obstetric trauma without instrument), were found to have a relatively small financial impact. However, other criteria (such as feasibility, availability of data, etc.) have, presumably, also guided the OECD’s selection of PSIs for country comparisons.

Contrasting our findings on the costs of the PSIs with the previous results of Zhan and Miller [14] shows various consistencies. According to Zhan and Miller [14], the highest excess costs per case were generated by the following five PSIs (in descending order of magnitude): PSI 13 (postoperative sepsis), PSI 10 (postoperative acute kidney injury), PSI 11 (postoperative respiratory failure), PSI 14 (wound dehiscence), and PSI 07 (CVC bloodstream infection). These were also the PSIs that showed the highest per case costs in our study. Zahn and Miller (2003) did not explicitly calculate the cumulative national costs of PSIs, but they did report the frequencies of each PSI, enabling us to calculate the cumulative costs ourselves based on their results. By doing so, we found that the following three PSIs had the highest national cost impact according to Zahn and Miller (in descending order of magnitude): PSI 03 (pressure ulcer), PSI 07 (CVC bloodstream infection), and PSI 12 (perioperative embolism or thrombosis). Our findings are consistent with these previous results regarding the high financial impact of PSI 12 (perioperative embolism or thrombosis) and PSI 07 (CVC bloodstream infection). However, according to our calculations, PSI 09 (postoperative hemorrhage/hematoma) is the most expensive PSI in Switzerland, while PSI 03 (pressure ulcer) is the most expensive in the US according to Zhan and Miller [14]. This disparity may arise from differing incidence rates of the PSIs in Switzerland and the US (see the next paragraph) but could also be the result of different treatment practices and coding incentives or differences in our medical coding systems (see below).

Comparing our findings on the frequency of the PSIs with the results of Zhan and Miller [14] reveals that although they also observed the obstetrical PSIs to be the most frequent, in absolute terms, they generally found higher incidence rates for most investigated PSIs. These differences may be because they used the earlier versions of PSI definitions that were currently in use in 2003. Since then, the PSIs have evolved considerably and now use adapted inclusion and exclusion criteria that have been improved on a continual basis over the last two decades. In fact, the transition from using ICD-9-CM to ICD-10-CM/PCS coding was a major transition for the PSI specifications over the last 20 years. In addition, several PSIs such as 09, 10, 12, and 15 underwent major redefinitions [20]. Furthermore, the medical coding practice (i.e., what is coded) and the coding quality (i.e., how well it is coded) have also been further developed since then. In addition, the fact that different coding systems (ICD-10-GM) are used in Switzerland and the US (ICD-9-CM at that time) may also have impacted the differing incidence rates in our study compared to the earlier results. Alternatively, it could be argued that the quality of care has improved over the last two decades, that healthcare quality is different between Switzerland and the US, or that the health status of the Swiss population is higher (as the US has higher rates of illnesses and lower life expectancy than Switzerland) [32]. However, it is similarly likely, in our opinion, that the PSIs are not as frequently coded in Switzerland due to different coding practices and/or incentives or because the PSIs are currently not used in annual healthcare quality assessments and, thus, may not be as closely monitored.

Furthermore, it must be noted that the order of magnitude of the associated excess costs of the PSIs between the per-event costs and the nationally aggregated costs did not only change due to the frequencies of the PSIs. Rather the relative financial impact of certain PSIs also decreased at a national level because we adjusted for the co-occurrence of different PSIs in our estimation of the national costs (see Section 2.3.3). This was particularly important for PSI 10 (postoperative acute kidney injury), PSI 11 (postoperative respiratory failure), and PSI 13 (postoperative sepsis), which often co-occurred. This makes sense from a clinical perspective because PSI 13 (postoperative sepsis) often precedes PSI 10 (postoperative acute kidney injury) and/or PSI 11 (postoperative respiratory failure) in cases of multiple organ system failure due to, for example, septic shock. Therefore, the adjustment for the co-occurrence of PSIs reduced the associated costs of these three PSIs at a national level compared to their per-event costs (which did not include such an adjustment).

### PSIs with a high risk for mortality and premature death of patients

Our study is, to the best of our knowledge, the first to provide information about the financial impact of PSI 02 (death in low-mortality DRGs) and PSI 04 (death after serious complications). We suppose that previous studies have not investigated these two PSIs because their outcome of interest is death, and they may have thus been assumed to decrease (rather than increase) costs due to a premature end of the hospitalization. However, we hypothesized that this need not necessarily be the case and included a variable for premature death as a covariate in our regression analyses to distinguish between patients that died prematurely (i.e., resulting in lower costs) and those that succumbed after several treatment attempts (i.e., resulting in higher costs). As hypothesized, our regression results revealed that costs only decrease in these PSI samples if patients deceased prematurely but, on the contrary, increase if they died at a later stage during the hospitalization. However, significantly higher costs and a trend towards higher costs associated with PSI 04 (death after serious complications) and PSI 02 (death in low-mortality DRGs), respectively, were already found in simple comparisons of means (without any correction for the proportion of patients that died prematurely). This demonstrates that, overall, costs increased for patients that died within these PSI samples. These findings support the notion that excess costs arise from trying to save patients [33]. Furthermore, the cost-decreasing effects associated with premature deaths were also observed across many other high-mortality PSI samples, such as PSI 06 (iatrogenic pneumothorax), PSI 07 (CVC bloodstream infection), PSI 10 (postoperative acute kidney injury), PSI 12 (perioperative embolism or thrombosis), and PSI 13 (postoperative sepsis). However, considered from a national perspective, the cost-reducing effect of premature deaths can be neglected across most PSIs given the rare occurrence of premature deaths in these PSIs.

### The practical relevance and limitations of our findings

Our study provides the most comprehensive examination of the excess costs associated with PSI-related adverse events to date. Most previous studies investigating the excess costs of PSIs either used individualized definitions or only examined a subset of PSIs. In contrast, we calculated the costs of all PSIs and compared their financial impact per case and on a nationally aggregated level. The total excess costs associated with all PSIs in Switzerland were estimated as roughly CHF 347 million in 2019, which is approximately 2.2% of the annual inpatient costs of CHF 15.7 billion [34]. Even though this is just an estimation, it demonstrates the potential for considerable national cost savings by reducing PSI-related adverse events. Interestingly, Hoogervorst-Schilp, Langelaan, Spreeuwenberg, De Bruijne and Wagner [35] found similar results in the Netherlands, where adverse events were estimated to produce approximately EUR 300 million in additional costs, corresponding to 1.3% of their national hospital costs.

It must, however, be highlighted that our study focused exclusively on the costs associated with adverse events covered by the PSIs. This includes only a fraction of all adverse event types and, therefore, does not allow for a conclusive statement concerning the potential cost savings across all in-hospital adverse events. Adverse drug reactions, for example, which are the second most common type of adverse events [36], are not covered by the PSIs. According to a German study [37], 1.7% of inpatient hospital costs result from adverse drug events. However, even if the costs of the PSIs only represent a fraction of the total excess costs of all in-hospital adverse events [14], their advantage is that they enable us to measure patient safety-adverse events consistently and allow for international comparisons.

The results presented in this study enable the prioritization of investments in particular aspects of patient safety that will result in the highest potential cost savings. For example, a 50% reduction in the frequency of the three most expensive adverse events, namely PSI 09 (postoperative hemorrhage/hematoma), PSI 04 (death after serious complications), and PSI 12 (perioperative embolism or thrombosis) could potentially save CHF 80 million per year in Switzerland. Our findings can thus help policymakers to select patient safety enhancing initiatives that, in addition to reducing suffering, also have the greatest potential for reducing national healthcare expenditure.

One significant limitation of our study is that we only considered costs that arose during inpatient stays. As has been pointed out [13], a substantial additional proportion (up to 20%) of the costs due to adverse events accrues in ambulatory settings after discharge. According to Mello, Studdert, Thomas, Yoon and Brennan [38], these additional costs could even be as high as 78%. Furthermore, we only included cases in our cost analyses that met the inclusion and exclusion criteria for the various PSI samples. These criteria often specifically exclude many cases at particularly high risk for adverse events in order to enable better stratification and more accurate hospital quality comparisons. As a result, a number of cases with additionally present PSI-related adverse events were neither included in our analyses nor in our estimation of the national costs due to such adverse events.

Another important limitation of our study is the fact that there currently exists no “present on admission” (POA) indicator for secondary diagnoses in Switzerland. This introduces an important bias that, presumably, increases the number of adverse events and, thus, the nationally aggregated costs in certain PSIs [39]. Based on previous research, this incidence inflating effect of false positives is most likely to occur in PSI 03 (pressure ulcer) and PSI 08 (fall with hip fracture), where POA information has shown the greatest benefits in terms of improving positive predictive values [40]. Another study identified 59% (in PSI 03) and 52% (in PSI 08) of cases flagged as an adverse event to be present on admission [41]. To assess the potential impact of POA-cases, we included an alternative calculation of the national cost projection in the supplemental material (Table S21 Table in S1 Appendix), which excludes an estimated percentage of POA-cases for each PSI (based on findings from previous research, see [41]). This reduced the aggregated national costs by approximately 14%. However, we believe that the cost inflating effect of POA-cases in some PSIs is compensated by underestimating PSI incidence due to coding inaccuracies across many of the PSIs. Previous research has shown that the total costs associated with adverse events appears to be underestimated by 15% because of coding errors [23] and this effect is likely to be even more pronounced in countries such as Switzerland, which have previously not systematically assessed the adverse events covered by the PSIs.

An important limitation to the generalization of our findings lies in the fact that we used a translation of the original PSI specifications from the US medical coding system (ICD-10-CM/PCS) into the Swiss coding system (ICD-10-GM and CHOP). Other peculiarities of the Swiss healthcare system may also decrease the generalization of our results. For instance, the fact that two separate hospitalizations with an intermediate early readmission within 18 days in the same major diagnostic category (MDC) are combined in the Swiss administrative data could change the reported rates of certain PSIs. PSIs such as PSI 09 (postoperative hemorrhage/hematoma) will also be registered if they occurred after discharge, which potentially increases the sensitivity of the respective PSIs. In addition, the presence of what was coined “nursing- or waiting patients” (receiving no treatment) in the Swiss administrative data required us to exclude these patients from analyses. Such particularities of the Swiss healthcare system limit comparability with previous research from countries with other healthcare settings.

An additional limitation stems from the fact that some of the PSIs have overlapping samples (e.g., PSI 04 with PSI 12 and PSI 13). We adjusted our results for this, but due to the relatively low sample sizes of several PSIs, it was not possible to fully separate all cases from different PSIs during the matching process. Thus, 20% and 6% of the matched controls of PSI 04 also belong to the sample of PSI 12 and PSI 13, respectively. Finally, by only examining total costs in our study, we cannot draw conclusions about who carries these costs in the healthcare system. More specifically, it is not certain if hospitals have to bear some of the excess costs associated with the adverse events or if the DRG reimbursements in Switzerland contain full compensation for these complications. Future research should investigate these issues to improve our understanding of the financial implications of in-hospital adverse events.

## Conclusion

Our study revealed that patients who experienced an adverse event covered by the PSIs were associated with CHF 27,409 higher costs, 7.8-day longer stays, 2.5 times more early readmissions, and 4.1 times higher mortality rates. Cumulatively, we estimated that roughly CHF 347 million excess costs are generated each year in relation to the investigated PSIs in Switzerland. However, the contribution of the different PSIs to these national costs varies markedly. Three PSIs alone account for nearly half of the national excess costs: PSI 09 (postoperative hemorrhage/hematoma), PSI 04 (death after serious complications), and PSI 12 (perioperative embolism or thrombosis), which are associated with costs of CHF 58, 57, and 55 million, respectively. Our results serve to inform the funding allocation decisions of policymakers and regulators by enabling them to consider the financial impact of these adverse events alongside the suffering they cause.

## Supporting information

S1 Appendix 1:3 matching (comparisons of means, number of premature deaths, individual regression analyses across all PSIs and estimation of the total costs of PSI-related adverse events). (DOCX)

S2 Appendix 1:1 matching (sample characteristics, mean comparisons, and an overview of excess costs). (DOCX)
