# Supplementary material for: Assessing the excess costs of the in-hospital adverse events covered by the AHRQ’s Patient Safety Indicators in Switzerland
Source: PLoS One. 2024 Feb 5;19(2):e0285285. doi: 10.1371/journal.pone.0285285 (PMC10843032; doi:10.1371/journal.pone.0285285)
Supplement: S1 Appendix — (DOCX) [file pone.0285285.s001.docx]

supplementary file 1

**Comparisons of means and relative frequencies in the variables used for the 1:3 matching:**

Table S1.1 Comparisons of means and relative frequencies across the matching variables

|  |  | **Age^a^** | | **Sex^b^** | | **Nationality** | | **Emergency** | | **Nursing home** | | **Transferred** | | **Elixhauser Index** | |
| --- | --- | --- | --- | --- | --- | --- | --- | --- | --- | --- | --- | --- | --- | --- | --- |
|  | **PSI** | **0** | **1** | **0** | **1** | **0** | **1** | **0** | **1** | **0** | **1** | **0** | **1** | **0** | **1** |
| PSI 02:  Death in low-mortality DRGs | Mean/RF | 76.59 | 77.56 | 49.61% | 51.16% | 79.84% | 79.07% | 79.07% | 74.42% | 14.73% | 16.28% | 8.53% | 11.63% | 7.25 | 6.98 |
|  | SD | 15.62 | 19.71 | - | - | - | - | - | - | - | - | - | - | 10.13 | 11.40 |
|  | test stat. | -0.91 | | 0.03 | | 0.01 | | 0.41 | | 0.06 | | 0.37 | | -0.42 | |
|  | p | .362 | | .860 | | .913 | | .524 | | .806 | | .551 | | .675 | |
| PSI 03:  Pressure ulcer ^d^ | Mean/RF | 77.82 | 78.35 | 53.03% | 53.53% | 81.73% | 80.92% | 82.08% | 82.89% | - | - | - | - | 15.43 | 17.46 |
|  | SD | 12.61 | 13.26 | - | - | - | - | - | - | - | - | - | - | 14.77 | 15.45 |
|  | test stat. | -1.79 | | 0.07 | | 0.28 | | 0.29 | | - | | - | | -3.44 | |
|  | p | .073 | | .798 | | .595 | | .589 | | - | | - | | .001 ** | |
| PSI 04:  Death after serious compli-cations | Mean/RF | 69.93 | 70.68 | 61.80% | 64.50% | 79.61% | 76.91% | 64.76% | 63.93% | 3.15% | 3.63% | 11.77% | 16.03% | 18.97 | 20.58 |
|  | SD | 14.20 | 14.68 | - | - | - | - | - | - | - | - | - | - | 15.40 | 15.77 |
|  | test stat. | -1.35 | | 1.22 | | 1.73 | | 0.12 | | 0.28 | | 6.36 | | -2.06 | |
|  | p | .177 | | .269 | | .189 | | .732 | | .597 | | .012 * | | .039 * | |
| PSI 05:  Retained surgical items | Mean/RF | 58.57 | 56.51 | 39.91% | 40.79% | 71.93% | 72.37% | 30.26% | 28.95% | 2.19% | 1.32% | 1.32% | 2.63% | 4.66 | 4.61 |
|  | SD | 18.55 | 18.92 | - | - | - | - | - | - | - | - | - | - | 10.76 | 11.45 |
|  | test stat. | -0.76 | | 0.02 | | 0.01 | | 0.05 | | 0.23 | | 0.61 | | -0.07 | |
|  | p | .448 | | .893 | | .941 | | .828 | | > .999 | | .602 | | .941 | |
| PSI 06:  Iatrogenic pneumo-thorax | Mean/RF | 68.36 | 67.13 | 49.57% | 49.68% | 84.19% | 83.55% | 53.66% | 49.68% | 2.37% | 1.61% | 10.86% | 12.58% | 13.42 | 13.84 |
|  | SD | 17.97 | 15.92 | - | - | - | - | - | - | - | - | - | - | 14.06 | 13.28 |
|  | test stat. | -1.81 | | 0.00 | | 0.07 | | 1.48 | | 0.62 | | 0.69 | | -1.06 | |
|  | p | .071 | | .974 | | .788 | | .224 | | .432 | | .407 | | .288 | |
| PSI 07:  CVC bloodstream infection | Mean/RF | 65.50 | 65.40 | 71.91% | 71.07% | 78.66% | 78.48% | 71.55% | 70.71% | 1.93% | 2.17% | 20.74% | 21.34% | 14.39 | 15.44 |
|  | SD | 20.35 | 15.74 | - | - | - | - | - | - | - | - | - | - | 14.75 | 14.64 |
|  | test stat. | -1.81 | | 0.15 | | 0.01 | | 0.14 | | 0.12 | | 0.09 | | -1.94 | |
|  | p | .070 | | .703 | | .928 | | .704 | | .725 | | .763 | | .053 | |
| PSI 08:  Fall with hip fracture | Mean/RF | 78.17 | 79.32 | 54.65% | 49.55% | 86.49% | 85.59% | 80.78% | 78.38% | 8.41% | 14.41% | 20.12% | 18.02% | 12.70 | 13.25 |
|  | SD | 10.77 | 12.09 | - | - | - | - | - | - | - | - | - | - | 13.91 | 12.78 |
|  | test stat. | -1.48 | | 0.87 | | 0.06 | | 0.30 | | 3.36 | | 0.23 | | -0.96 | |
|  | p | .139 | | .350 | | .811 | | .582 | | .067 | | .629 | | .336 | |
| PSI 09: Post-operative hemorrhage/hematoma | Mean/RF | 60.12 | 59.63 | 55.63% | 56.86% | 77.33% | 77.22% | 26.49% | 27.02% | 0.92% | 0.96% | 5.34% | 5.26% | 6.29 | 6.87 |
|  | SD | 18.59 | 19.68 | - | - | - | - | - | - | - | - | - | - | 11.48 | 11.50 |
|  | test stat. | -0.43 | | 1.27 | | 0.01 | | 0.29 | | 0.03 | | 0.02 | | -3.74 | |
|  | p | .667 | | .260 | | .905 | | .590 | | .863 | | .882 | | < .001 *** | |
| PSI 10:  Post-operative acute kidney injury | Mean/RF | 70.43 | 68.69 | 68.48% | 66.82% | 71.43% | 72.35% | 3.42% | 2.76% | 0.00% | 0.00% | 8.07% | 9.68% | 30.44 | 31.89 |
|  | SD | 13.53 | 13.39 | - | - | - | - | - | - | - | - | - | - | 16.03 | 16.32 |
|  | test stat. | -1.67 | | 0.21 | | 0.07 | | 0.22 | | - | | 0.54 | | -1.01 | |
|  | p | .095 | | .651 | | .794 | | .640 | | - | | .464 | | .311 | |
| PSI 11:  Post-operative respiratory failure | Mean/RF | 69.77 | 67.05 | 82.95% | 82.95% | 83.33% | 79.55% | 2.27% | 2.27% | 1.52% | 1.14% | 4.55% | 5.68% | 25.23 | 25.60 |
|  | SD | 15.53 | 11.54 | - | - | - | - | - | - | - | - | - | - | 17.68 | 18.05 |
|  | test stat. | -2.59 | | 0.00 | | 0.65 | | 0.00 | | 0.07 | | 0.19 | | -0.13 | |
|  | p | .010 * | | > .999 | | .419 | | > .999 | | > .999 | | .774 | | .894 | |
| PSI 12:  Peri-operative embolism or thrombosis | Mean/RF | 70.21 | 69.16 | 52.44% | 53.35% | 82.91% | 82.94% | 56.16% | 54.64% | 3.58% | 3.17% | 11.91% | 13.03% | 17.73 | 19.39 |
|  | SD | 15.87 | 15.40 | - | - | - | - | - | - | - | - | - | - | 16.37 | 16.38 |
|  | test stat. | -2.79 | | 0.35 | | 0.00 | | 0.97 | | 0.52 | | 1.23 | | -3.74 | |
|  | p | .005 ** | | .556 | | .978 | | .325 | | .470 | | .268 | | < .001 *** | |
| PSI 13:  Post-operative sepsis | Mean/RF | 71.47 | 70.18 | 68.82% | 66.24% | 80.58% | 80.17% | 1.41% | 1.48% | 1.20% | 1.27% | 4.93% | 6.96% | 22.54 | 23.33 |
|  | SD | 13.29 | 13.28 | - | - | - | - | - | - | - | - | - | - | 16.12 | 16.33 |
|  | test stat. | -1.59 | | 1.09 | | 0.04 | | 0.01 | | 0.01 | | 2.87 | | -0.94 | |
|  | p | .111 | | .296 | | .846 | | .912 | | .905 | | .090 | | .347 | |
| PSI 14:  Wound dehiscence | Mean/RF | 70.36 | 69.77 | 66.67% | 66.23% | 80.57% | 82.78% | 51.66% | 49.01% | 1.55% | 0.66% | 6.18% | 8.61% | 12.85 | 14.82 |
|  | SD | 13.91 | 13.59 | - | - | - | - | - | - | - | - | - | - | 14.64 | 13.24 |
|  | test stat. | -0.47 | | 0.01 | | 0.36 | | 0.32 | | 0.68 | | 1.06 | | -2.14 | |
|  | p | .642 | | .921 | | .548 | | .573 | | .686 | | .304 | | .032 | |
| PSI 18:  Obstetric trauma with instrument ^e^ | Mean/RF | 33.27 | 33.12 | - | - | 55.95% | 55.54% | 56.19% | 56.43% | - | - | 0.48% | 0.71% | -0.59 | -0.59 |
|  | SD | 4.96 | 4.75 | - | - | - | - | - | - | - | - | - | - | 2.88 | 2.91 |
|  | test stat. | -0.61 | | - | | 0.03 | | 0.01 | | - | | 0.45 | | -0.24 | |
|  | p | .544 | | - | | .863 | | .922 | | - | | .509 | | .808 | |
| PSI 19:  Obstetric trauma without instrument ^e^ | Mean/RF | 32.62 | 32.64 | - | - | 59.08% | 59.04% | 55.82% | 56.14% | - | - | 0.16% | 0.36% | -0.53 | -0.57 |
|  | SD | 4.88 | 4.77 | - | - | - | - | - | - | - | - | - | - | 2.81 | 2.73 |
|  | test stat. | -0.08 | | - | | 0.00 | | 0.03 | | - | | 1.19 | | -0.27 | |
|  | p | .935 | | - | | .984 | | .872 | | - | | .377 | | .784 | |
|  |  | Age (G)^c^ | | Sex | | Nationality: Swiss | | Birth weight | | Head size | | No. prev. live births^f^ | | Elixhauser Index | |
|  |  | 0 | 1 | 0 | 1 | 0 | 1 | 0 | 1 | 0 | 1 | 0 | 1 | 0 | 1 |
| PSI 17:  Birth traum | Mean/RF | 0.01 | -0.03 | 64.10% | 67.95% | 50.43% | 51.28% | 3469.44 | 3460.05 | 34.17 | 34.09 | 0.31 | 0.32 | 0.21 | 0.42 |
|  | SD | 0.98 | 1.07 | - | - | - | - | 549.27 | 582.28 | 5.74 | 5.78 | 0.55 | 0.65 | 1.36 | 1.99 |
|  | test stat. | -0.09 | | 0.38 | | 0.02 | | -0.36 | | -0.23 | | -0.55 | | -0.89 | |
|  | p | .931 | | .537 | | .896 | | .715 | | .819 | | .584 | | .374 | |
| *Note*. *** = *p* < .001; ** = *p* < 0.01; * = *p* < 0.05. Binary variables are expressed as relative frequencies (RF) in percent (%). Continuous variables are expressed as means and standard deviations (SD). ^a^ Rounded up to 5 years. ^b^ 1 = male. ^c^ Gestational age. Gestational age is measured in weeks, but it was standardized to facilitate the interpretation. ^d^ In PSI 3, patients were excluded from the sample if they were transferred from another hospital or were admitted from a nursing home. ^e^ The variables sex and admission from a nursing home were excluded in PSIs 18 and 19 because of missing relevance. *p* probability value. ^f^ Number of all previous live births of the mother. Please note that the matching variable “emergency” consists of cases with either admission type being indicated as “emergency” or admitted by being recorded as “ambulance”. That is the reason why PSIs 10, 11, and 13 have a small percentage of non-negative values in this matching variable, despite the fact that these PSIs focus on cases with admission type equal to elective. | | | | | | | | | | | | | | | |

The number of premature deaths across all PSIs:

Table S1.2 Number of premature deaths in PSI-flagged cases in the matched data

|  |  | **Matched data** | | |
| --- | --- | --- | --- | --- |
|  |  | **Flagged cases** | **Premature deaths** | **% premature deaths** |
| PSI 02 | Death in low-mortality DRGs | 43 | 2 | 4.65% |
| PSI 03 | Pressure ulcer | 865 | 0 | 0.00% |
| PSI 04 | Death after serious complications | 524 | 104 | 19.85% |
| PSI 05 | Retained surgical items | 76 | 0 | 0.00% |
| PSI 06 | Iatrogenic pneumothorax | 310 | 8 | 2.58% |
| PSI 07 | CVC bloodstream infection | 553 | 60 | 10.85% |
| PSI 08 | Fall with hip fracture | 111 | 1 | 0.90% |
| PSI 09 | Postoperative hemorrhage/hematoma | 2717 | 14 | 0.52% |
| PSI 10 | Postoperative acute kidney injury | 217 | 14 | 6.45% |
| PSI 11 | Postoperative respiratory failure | 88 | 1 | 1.14% |
| PSI 12 | Perioperative embolism or thrombosis | 1389 | 26 | 1.87% |
| PSI 13 | Postoperative sepsis | 474 | 10 | 2.11% |
| PSI 14 | Wound dehiscence | 151 | 0 | 0.00% |
| PSI 15 | Accidental punctures or lacerations |  |  |  |
| PSI 17 | Birth trauma | 78 | 0 | 0.00% |
| PSI 18 | Obstetric trauma with instrument | 560 | 0 | 0.00% |
| PSI 19 | Obstetric trauma without instrument | 830 | 0 | 0.00% |

Individual regression analyses across all PSIs:

**Table S2** Regression analysis using the occurrence of PSI 02 (death in low-mortality DRGs) and several covariates to explain total costs (in CHF)

|  | ***B (in CHF)*** | ***Std. Error*** | ***t*** | ***p*** |  | |
| --- | --- | --- | --- | --- | --- | --- |
| Constant | 17,981.11 | 4,335.57 | 4.147 | < .001 | ******* | |
| PSI 02 occurrence | 7,333.19 | 1,877.50 | 3.906 | < .001 | ******* | |
| Age | -24.60 | 53.33 | -0.461 | .645 |  | |
| Sex: male | -2,532.50 | 1,630.18 | -1.554 | .122 |  | |
| Nationality: Swiss | -1,719.14 | 2,061.08 | -0.834 | .405 |  | |
| Admission: Emergency | -7,815.18 | 2,072.80 | -3.770 | < .001 | ******* | |
| Admission: From nursing home | -2,425.66 | 2,345.60 | -1.034 | .303 |  | |
| Admission: Transferred | -1,450.00 | 2,855.70 | -0.508 | .612 |  | |
| Elixhauser Index | 362.26 | 83.25 | 4.351 | < .001 | ******* | |
| Premature death | -14,774.58 | 7,921.39 | -1.865 | .064 |  | |
| *Note*. *** = *p* < .001; ** = *p* < 0.01; * = *p* < 0.05; *B* unstandardized beta coefficients (costs), *t* test statistic, *p* probability value, *CHF* Swiss francs. | | | | | |  |

Table S3 Regression analysis using the occurrence of PSI 03 (pressure ulcer) and several covariates to explain total costs (in CHF)

|  | ***B (in CHF)*** | ***Std. Error*** | ***t*** | ***p*** |  |
| --- | --- | --- | --- | --- | --- |
| Constant | 58,990.75 | 5,285.83 | 11.160 | < .001 | ******* |
| PSI 03 occurrence | 28,642.17 | 1,807.62 | 15.845 | < .001 | ******* |
| Age | -562.33 | 62.89 | -8.941 | < .001 | ******* |
| Sex: male | 3,187.70 | 1,593.49 | 2.000 | .046 | ***** |
| Nationality: Swiss | -1,248.64 | 2,041.24 | -0.612 | .541 |  |
| Admission: Emergency | -3,765.00 | 2,068.61 | -1.820 | .069 |  |
| Elixhauser Index | 609.61 | 52.70 | 11.567 | < .001 | ******* |
| Premature death | 57,897.99 | 32,530.15 | 1.780 | .075 |  |
| *Note*. *** = *p* < .001; ** = *p* < 0.01; * = *p* < 0.05; *B* unstandardized beta coefficients (costs), *t* test statistic, *p* probability value, *CHF* Swiss francs. In PSI 3, patients were excluded from the sample if they were transferred from another hospital or were admitted from a nursing home. Because of this, these two variables are not included here. | | | | | |

Table S4 Regression analysis using the occurrence of PSI 04 (death after serious complications) and several covariates to explain total costs (in CHF)

|  | ***B (in CHF)*** | ***Std. Error*** | ***t*** | ***p*** |  |
| --- | --- | --- | --- | --- | --- |
| Constant | 171,222.63 | 14,200.94 | 12.057 | < .001 | ******* |
| PSI 04 occurrence | 27,962.38 | 6,152.62 | 4.545 | < .001 | ******* |
| Age | -1,723.89 | 176.90 | -9.745 | < .001 | ******* |
| Sex: male | 7,704.53 | 5,069.15 | 1.520 | .129 |  |
| Nationality: Swiss | -10,578.35 | 6,123.29 | -1.728 | .084 |  |
| Admission: Emergency | 7,686.42 | 5,170.27 | 1.487 | .137 |  |
| Admission: From nursing home | -11,476.32 | 13,980.21 | -0.821 | .412 |  |
| Admission: Transferred | 30,698.19 | 7,411.42 | 4.142 | < .001 | ******* |
| Elixhauser Index | 1,612.22 | 159.15 | 10.130 | < .001 | ******* |
| Premature death | -77,166.33 | 12,229.28 | -6.310 | < .001 | ******* |
| *Note*. *** = *p* < .001; ** = *p* < 0.01; * = *p* < 0.05; *B* unstandardized beta coefficients (costs), *t* test statistic, *p* probability value, *CHF* Swiss francs. | | | | | |

Table S5 Regression analysis using the occurrence of PSI 05 (retained surgical item) and several covariates to explain total costs (in CHF)

|  | ***B (in CHF)*** | ***Std. Error*** | ***t*** | ***p*** |  |
| --- | --- | --- | --- | --- | --- |
| Constant | 5,366.66 | 5,826.11 | 0.921 | .358 |  |
| PSI 05 occurrence | 16,303.89 | 3,599.20 | 4.530 | < .001 | ******* |
| Age | 9.50 | 91.12 | 0.104 | .917 |  |
| Sex: male | 6,479.09 | 3,232.65 | 2.004 | .046 | ***** |
| Nationality: Swiss | 944.26 | 3,552.98 | 0.266 | .791 |  |
| Admission: Emergency | 430.82 | 3,539.75 | 0.122 | .903 |  |
| Admission: From nursing home | -2,295.13 | 11,586.47 | -0.198 | .843 |  |
| Admission: Transferred | 35,857.14 | 12,344.78 | 2.905 | .004 | ****** |
| Elixhauser Index | 906.87 | 155.62 | 5.828 | < .001 | ******* |
| Premature death | -7,353.27 | 27,406.66 | -0.268 | .789 |  |
| *Note*. *** = *p* < .001; ** = *p* < 0.01; * = *p* < 0.05; *B* unstandardized beta coefficients (costs), *t* test statistic, *p* probability value, *CHF* Swiss francs. | | | | | |

Table S6 Regression analysis using the occurrence of PSI 06 (iatrogenic pneumothorax) and several covariates to explain total costs (in CHF)

|  | ***B (in CHF)*** | ***Std. Error*** | ***t*** | ***p*** |  |
| --- | --- | --- | --- | --- | --- |
| Constant | 5,366.66 | 5,826.11 | 0.921 | .358 |  |
| PSI 06 occurrence | 25,405.86 | 2,581.84 | 9.840 | < .001 | ******* |
| Age | -65.37 | 66.50 | -0.983 | .326 |  |
| Sex: male | 1,422.02 | 2,253.18 | 0.631 | .528 |  |
| Nationality: Swiss | -7,367.24 | 3,081.84 | -2.391 | .017 | ***** |
| Admission: Emergency | 4,509.32 | 2,317.18 | 1.946 | .052 |  |
| Admission: From nursing home | -1,806.42 | 7,686.99 | -0.235 | .814 |  |
| Admission: Transferred | 17,896.63 | 3,573.42 | 5.008 | < .001 | ******* |
| Elixhauser Index | 446.18 | 83.72 | 5.329 | < .001 | ******* |
| Premature death | -28,230.22 | 11,479.41 | -2.459 | .014 | ***** |
| *Note*. *** = *p* < .001; ** = *p* < 0.01; * = *p* < 0.05; *B* unstandardized beta coefficients (costs), *t* test statistic, *p* probability value, *CHF* Swiss francs. | | | | | |

Table S7 Regression analysis using the occurrence of PSI 07 (CVC bloodstream infection) and several covariates to explain total costs (in CHF)

|  | ***B (in CHF)*** | ***Std. Error*** | ***t*** | ***p*** |  |
| --- | --- | --- | --- | --- | --- |
| Constant | 28,141.17 | 6,177.19 | 4.556 | < .001 | ******* |
| PSI 07 occurrence | 69,462.52 | 3,266.90 | 21.263 | < .001 | ******* |
| Age | -508.07 | 79.54 | -6.388 | < .001 | ******* |
| Sex: male | 2,317.93 | 3,162.54 | 0.733 | .464 |  |
| Nationality: Swiss | -4,008.47 | 3,513.54 | -1.141 | .254 |  |
| Admission: Emergency | 6,791.05 | 3,277.13 | 2.072 | .038 | ***** |
| Admission: From nursing home | -14,957.76 | 10,234.20 | -1.462 | .144 |  |
| Admission: Transferred | 18,179.77 | 3,654.25 | 4.975 | < .001 | ******* |
| Elixhauser Index | 1,641.73 | 105.40 | 15.576 | < .001 | ******* |
| Premature death | -33,145.95 | 16,827.54 | -1.970 | .049 | ***** |
| *Note*. *** = *p* < .001; ** = *p* < 0.01; * = *p* < 0.05; *B* unstandardized beta coefficients (costs), *t* test statistic, *p* probability value, *CHF* Swiss francs. | | | | | |

Table S8 Regression analysis using the occurrence of PSI 08 (fall with hip fracture) and several covariates to explain total costs (in CHF)

|  | ***B (in CHF)*** | ***Std. Error*** | ***t*** | ***p*** |  |
| --- | --- | --- | --- | --- | --- |
| Constant | 75,683.45 | 16,970.60 | 4.460 | < .001 | ******* |
| PSI 08 occurrence | 30,607.06 | 4,719.56 | 6.485 | < .001 | ******* |
| Age | -785.74 | 195.07 | -4.028 | < .001 | ******* |
| Sex: male | 1,626.02 | 4,192.63 | 0.388 | .698 |  |
| Nationality: Swiss | -6,638.75 | 6,025.11 | -1.102 | .271 |  |
| Admission: Emergency | 5,013.03 | 5,238.91 | 0.957 | .339 |  |
| Admission: From nursing home | -283.81 | 7,225.27 | -0.039 | .969 |  |
| Admission: Transferred | 10,442.57 | 5,285.19 | 1.976 | .049 | ***** |
| Elixhauser Index | 1,552.75 | 152.52 | 10.181 | < .001 | ******* |
| Premature death | -19,681.05 | 21,697.72 | -0.907 | .365 |  |
| *Note*. *** = *p* < .001; ** = *p* < 0.01; * = *p* < 0.05; *B* unstandardized beta coefficients (costs), *t* test statistic, *p* probability value, *CHF* Swiss francs. | | | | | |

Table S9 Regression analysis using the occurrence of PSI 09 (postoperative hemorrhage/hematoma) and several covariates to explain total costs (in CHF)

|  | ***B (in CHF)*** | ***Std. Error*** | ***t*** | ***p*** |  |
| --- | --- | --- | --- | --- | --- |
| Constant | 5,797.75 | 1,190.02 | 4.872 | < .001 | ******* |
| PSI 09 occurrence | 17,073.58 | 717.78 | 23.787 | < .001 | ******* |
| Age | 65.23 | 17.76 | 3.674 | < .001 | ******* |
| Sex: male | 828.77 | 628.28 | 1.319 | .187 |  |
| Nationality: Swiss | -1,710.51 | 754.91 | -2.266 | .023 | ***** |
| Admission: Emergency | 10,076.47 | 717.67 | 14.040 | < .001 | ******* |
| Admission: From nursing home | -4,713.64 | 3,270.21 | -1.441 | .150 |  |
| Admission: Transferred | 16,964.77 | 1,405.81 | 12.068 | < .001 | ******* |
| Elixhauser Index | 918.18 | 28.78 | 31.901 | < .001 | ******* |
| Premature death | -5,051.83 | 5,290.92 | -0.955 | .340 |  |
| *Note*. *** = *p* < .001; ** = *p* < 0.01; * = *p* < 0.05; *B* unstandardized beta coefficients (costs), *t* test statistic, *p* probability value, *CHF* Swiss francs. | | | | | |

Table S10 Regression analysis using the occurrence of PSI 10 (postoperative acute kidney injury) and several covariates to explain total costs (in CHF)

|  | ***B (in CHF)*** | ***Std. Error*** | ***t*** | ***p*** |  |
| --- | --- | --- | --- | --- | --- |
| Constant | 73,272.93 | 18,658.81 | 3.927 | < .001 | ******* |
| PSI 10 occurrence | 137,967.66 | 7,774.29 | 17.747 | < .001 | ******* |
| Age | -1,240.80 | 253.44 | -4.896 | < .001 | ******* |
| Sex: male | 7,403.49 | 7,132.42 | 1.038 | .300 |  |
| Nationality: Swiss | 582.15 | 7,511.07 | 0.078 | .938 |  |
| Admission: Emergency | -5,810.09 | 21,400.45 | -0.271 | .786 |  |
| Admission: Transferred | 24,395.65 | 13,677.90 | 1.784 | .075 |  |
| Elixhauser Index | 1,597.74 | 209.67 | 7.620 | < .001 | ******* |
| Premature death | -71,816.65 | 23,598.13 | -3.043 | .002 | ****** |
| *Note*. *** = *p* < .001; ** = *p* < 0.01; * = *p* < 0.05; *B* unstandardized beta coefficients (costs), *t* test statistic, *p* probability value, *CHF* Swiss francs. The variable admission from nursing home is not included here because there were no admissions from nursing homes within the PSI 10 cases of this sample. Please also note that the covariate “emergency” consists of cases with either admission type being indicated as “emergency” or admitted by being recorded as “ambulance”. That is the reason why PSI 10 has a small percentage of non-negative values in this covariate, despite the fact that this PSI focuses on cases with admission type equal to elective. | | | | | |

Table S11 Regression analysis using the occurrence of PSI 11 (postoperative respiratory failure) and several covariates to explain total costs (in CHF)

|  | ***B (in CHF)*** | ***Std. Error*** | ***t*** | ***p*** |  | |
| --- | --- | --- | --- | --- | --- | --- |
| Constant | 11,040.48 | 17,183.94 | 0.642 | .521 |  | |
| PSI 11 occurrence | 104,000.42 | 6,981.65 | 14.896 | < .001 | *** | |
| Age | -61.30 | 211.23 | -0.290 | .7772 |  | |
| Sex: male | 1,603.28 | 8,111.93 | 0.198 | .843 |  | |
| Nationality: Swiss | -11,947.67 | 7,972.36 | -1.499 | .135 |  | |
| Admission: emergency | 3,050.86 | 22,923.88 | 0.133 | .894 |  | |
| Admission: From nursing home | -14,938.98 | 26,597.16 | -0.562 | .575 |  | |
| Admission: transferred | 21,111.39 | 15,587.01 | 1.354 | .176 |  | |
| Elixhauser Index | 1,452.93 | 174.23 | 8.339 | < .001 | *** | |
| Premature death | -50,913.25 | 34,654.90 | -1.469 | .143 |  | |
| *Note*. *** = *p* < .001; ** = *p* < 0.01; * = *p* < 0.05; *B* unstandardized beta coefficients (costs), *t* test statistic, *p* probability value, *CHF* Swiss francs. Please also note that the covariate “emergency” consists of cases with either admission type being indicated as “emergency” or admitted by being recorded as “ambulance”. That is the reason why PSI 11 has a small percentage of non-negative values in this covariate, despite the fact that this PSI focuses on cases with admission type equal to elective. | | | | | |  |

Table S12 Regression analysis using the occurrence of PSI 12 (perioperative embolism or thrombosis) and several covariates to explain total costs (in CHF)

|  | ***B (in CHF)*** | ***Std. Error*** | ***t*** | ***p*** |  | |
| --- | --- | --- | --- | --- | --- | --- |
| Constant | 35,782.02 | 4,970.14 | 7.199 | < .001 | ******* | |
| PSI 12 occurrence | 32,815.55 | 2,219.60 | 14.784 | < .001 | ******* | |
| Age | -540.54 | 64.29 | -8.408 | < .001 | ******* | |
| Sex: male | 7,327.68 | 1,950.19 | 3.757 | < .001 | ******* | |
| Nationality: Swiss | -2,771.81 | 2,592.60 | -1.069 | .285 |  | |
| Admission: Emergency | 16,092.59 | 1,955.37 | 8.230 | < .001 | ******* | |
| Admission: From nursing home | -5,015.80 | 5,394.79 | -0.930 | .353 |  | |
| Admission: Transferred | 28,352.01 | 2,970.41 | 9.545 | < .001 | ******* | |
| Elixhauser Index | 1,408.06 | 60.38 | 23.319 | < .001 | ******* | |
| Premature death | -33,012.46 | 8,443.48 | -3.910 | < .001 | ******* | |
| *Note*. *** = *p* < .001; ** = *p* < 0.01; * = *p* < 0.05; *B* unstandardized beta coefficients (costs), *t* test statistic, *p* probability value, *CHF* Swiss francs. | | | | | |  |

Table S13 Regression analysis using the occurrence of PSI 13 (postoperative sepsis) and several covariates to explain total costs (in CHF)

|  | ***B (in CHF)*** | ***Std. Error*** | ***t*** | ***p*** |  | |
| --- | --- | --- | --- | --- | --- | --- |
| Constant | 46,403.55 | 10,186.56 | 4.555 | < .001 | *** | |
| PSI 13 occurrence | 91,568.15 | 4,064.36 | 22.530 | < .001 | *** | |
| Age | -696.22 | 135.25 | -5.148 | < .001 | *** | |
| Sex: male | 5,558.30 | 3,774.34 | 1.473 | .141 |  | |
| Nationality: Swiss | -3,487.24 | 4,474.11 | -0.779 | .436 |  | |
| Admission: emergency | -23,699.87 | 15,985.00 | -1.483 | .138 |  | |
| Admission: From nursing home | 7,608.12 | 16,307.08 | 0.467 | .641 |  | |
| Admission: Transferred | 37,545.39 | 8,282.39 | 4.533 | < .001 | *** | |
| Elixhauser Index | 1,435.09 | 109.06 | 13.159 | < .001 | *** | |
| Premature death | -42,874.46 | 20,560.97 | -2.085 | .037 | * | |
| *Note*. *** = *p* < .001; ** = *p* < 0.01; * = *p* < 0.05; *B* unstandardized beta coefficients (costs), *t* test statistic, *p* probability value, *CHF* Swiss francs. Please also note that the covariate “emergency” consists of cases with either admission type being indicated as “emergency” or admitted by being recorded as “ambulance”. That is the reason why PSI 13 has a small percentage of non-negative values in this covariate, despite the fact that this PSI focuses on cases with admission type equal to elective. | | | | | |  |

Table S14 Regression analysis using the occurrence of PSI 14 (wound dehiscence) and several covariates to explain total costs (in CHF)

|  | ***B (in CHF)*** | ***Std. Error*** | ***t*** | ***p*** |  |
| --- | --- | --- | --- | --- | --- |
| Constant | 28,776.95 | 12,005.43 | 2.397 | .017 | ***** |
| PSI 14 occurrence | 41,283.12 | 4,910.31 | 8.407 | < .001 | ******* |
| Age | -165.18 | 161.40 | -1.023 | .307 |  |
| Sex: male | 276.12 | 4,518.86 | 0.061 | .951 |  |
| Nationality: Swiss | -5,213.13 | 5,544.65 | -0.940 | .347 |  |
| Admission: Emergency | 10,442.61 | 4,310.56 | 2.423 | .016 | ***** |
| Admission: From nursing home | -12,348.72 | 18,901.80 | -0.653 | .514 |  |
| Admission: Transferred | 58,325.40 | 8,504.57 | 6.858 | < .001 | ******* |
| Elixhauser Index | 939.38 | 153.02 | 6.139 | < .001 | ******* |
| Premature death | -25,100.83 | 52,387.66 | -0.479 | .632 |  |
| *Note*. *** = *p* < .001; ** = *p* < 0.01; * = *p* < 0.05; *B* unstandardized beta coefficients (costs), *t* test statistic, *p* probability value, *CHF* Swiss francs. | | | | | |

Table S15 Regression analysis using the occurrence of PSI 17 (birth trauma) and several covariates to explain total costs (in CHF)

|  | ***B (in CHF)*** | ***Std. Error*** | ***t*** | ***p*** |  |
| --- | --- | --- | --- | --- | --- |
| Constant | 77,072.28 | 18,239.37 | 4.226 | < .001 | ******* |
| PSI 17 occurrence | 5,819.57 | 1,876.75 | 3.101 | .002 | ****** |
| Sex: male | 1,712.02 | 1,725.77 | 0.992 | .322 |  |
| Nationality: Swiss | -2,795.70 | 1,635.11 | -1.710 | .088 |  |
| Admission: Transferred | -2,791.75 | 5,505.73 | -0.507 | .612 |  |
| Elixhauser Index | 3,691.46 | 531.19 | 6.949 | < .001 | ******* |
| No. previous live births | -206.42 | 1,463.11 | -0.141 | .888 |  |
| Birth weight | -1,102.91 | 968.49 | -1.139 | .256 |  |
| Gestational age | -3,158.46 | 937.02 | -3.371 | .001 | ****** |
| Head size | 500.02 | 846.77 | 0.591 | .555 |  |
| *Note*. *** = *p* < .001; ** = *p* < 0.01; * = *p* < 0.05; *B* unstandardized beta coefficients (costs), *t* test statistic, *p* probability value, *CHF* Swiss francs. Gestational age is measured in weeks. Birth weight, gestational age, and head size were standardized to facilitate the interpretation. No patients died; therefore, we did not control for premature deaths. | | | | | |

Table S16 Regression analysis using the occurrence of PSI 18 (obstetric trauma with instrument) and several covariates to explain total costs (in CHF)

|  | ***B (in CHF)*** | ***Std. Error*** | ***t*** | ***p*** |  | |
| --- | --- | --- | --- | --- | --- | --- |
| Constant | 7,868.90 | 519.11 | 15.158 | < .001 | ******* | |
| PSI 18 occurrence | 1,211.15 | 170.37 | 7.109 | < .001 | ******* | |
| Age | 20.69 | 15.15 | 1.365 | .172 |  | |
| Nationality: Swiss | -187.82 | 149.51 | -1.256 | .209 |  | |
| Admission: Emergency | -429.82 | 148.95 | -2.886 | .004 | ****** | |
| Admission: Transferred | -1,225.85 | 1,013.01 | -1.210 | .226 |  | |
| Elixhauser Index | 15.98 | 25.60 | 0.624 | .533 |  | |
| *Note*. *** = *p* < .001; ** = *p* < 0.01; * = *p* < 0.05; *B* unstandardized beta coefficients (costs), *t* test statistic, *p* probability value, *CHF* Swiss francs. No patients died; therefore, we did not control for premature deaths. Similarly, the variables sex and admission from nursing home are not included here because no patients were male or admitted from a nursing home. | | | | | |  |

Table S17 Regression analysis using the occurrence of PSI 19 (obstetric trauma without instrument) and several covariates to explain total costs (in CHF)

|  | ***B (in CHF)*** | ***Std. Error*** | ***t*** | ***p*** |  |
| --- | --- | --- | --- | --- | --- |
| Constant | 7,275.55 | 303.20 | 23.996 | < .001 | ******* |
| PSI 19 occurrence | 1,440.25 | 100.38 | 14.349 | < .001 | ******* |
| Age | -18.40 | 8.99 | -2.047 | .041 | ***** |
| Nationality: Swiss | -50.89 | 88.90 | -0.572 | .567 |  |
| Admission: Emergency | -403.74 | 87.73 | -4.602 | < .001 | ******* |
| Admission: Transferred | -1,192.51 | 947.95 | -1.258 | .208 |  |
| Elixhauser Index | 4.30 | 15.59 | 0.276 | .782 |  |
| *Note*. *** = *p* < .001; ** = *p* < 0.01; * = *p* < 0.05; *B* unstandardized beta coefficients (costs), *t* test statistic, *p* probability value, *CHF* Swiss francs. No patients died; therefore, we did not control for premature deaths. Similarly, the variables sex and admission from nursing home are not included here because no patients were male or admitted from a nursing home. | | | | | |

**Table S18** Overview of the excess costs of PSI-related adverse events from the individual regression analyses presented above

|  |  | ***B (in CHF)*** | ***Std. Error*** | ***t*** | ***p*** |  |
| --- | --- | --- | --- | --- | --- | --- |
| PSI 02 | Death in low-mortality DRGs | 7,333.19 | 1,877.50 | 3.906 | < .001 | *** |
| PSI 03 | Pressure ulcer | 28,642.17 | 1,807.62 | 15.845 | < .001 | *** |
| PSI 04 | Death after serious complications | 27,962.38 | 6,152.62 | 4.545 | < .001 | *** |
| PSI 05 | Retained surgical items | 16,303.89 | 3,599.20 | 4.530 | < .001 | *** |
| PSI 06 | Iatrogenic pneumothorax | 25,405.86 | 2,581.84 | 9.840 | < .001 | *** |
| PSI 07 | CVC bloodstream infection | 69,462.52 | 3,266.90 | 21.263 | < .001 | *** |
| PSI 08 | Fall with hip fracture | 30,607.06 | 4,719.56 | 6.485 | < .001 | *** |
| PSI 09 | Perioperative hemorrhage/hematoma | 17,073.58 | 717.78 | 23.787 | < .001 | *** |
| PSI 10 | Postoperative acute kidney injury | 137,967.66 | 7,774.29 | 17.747 | < .001 | *** |
| PSI 11 | Postoperative respiratory failure | 104,000.42 | 6,981.65 | 14.896 | < .001 | *** |
| PSI 12 | Perioperative embolism or thrombosis | 32,815.55 | 2,219.60 | 14.784 | < .001 | *** |
| PSI 13 | Postoperative sepsis | 91,568.15 | 4,064.36 | 22.530 | < .001 | *** |
| PSI 14 | Wound dehiscence | 41,283.12 | 4,910.31 | 8.407 | < .001 | *** |
| PSI 17 | Birth trauma | 5,819.57 | 1,876.75 | 3.101 | .002 | ** |
| PSI 18 | Obstetric trauma with instrument | 1,211.15 | 170.37 | 7.109 | < .001 | *** |
| PSI 19 | Obstetric trauma without instrument | 1,440.25 | 100.38 | 14.349 | < .001 | *** |
| *Note*. *** = *p* < .001; ** = *p* < 0.01; *B* unstandardized beta coefficients (costs), *t* test statistic, *p* probability value, *CHF* Swiss francs. | | | | | |  |

Estimation of the total national costs of PSI-related adverse events in Switzerland:

Table S19 Regression analysis using the occurrence of all non-obstetrical PSIs and several covariates to explain total costs (in CHF)

|  |  | ***B (in CHF)*** | ***Std. Error*** | ***t*** | ***p*** |  |
| --- | --- | --- | --- | --- | --- | --- |
| Constant | | 13,795.93 | 989.81 | 13.938 | < .001 | *** |
| PSI 02 | Death in low-mortality DRGs | -9,015.84 | 7,621.49 | -1.183 | 0.237 |  |
| PSI 03 | Pressure ulcer | 21,037.08 | 1,761.53 | 11.943 | < .001 | *** |
| PSI 04 | Death after serious complications | 43,118.33 | 1,810.40 | 23.817 | < .001 | *** |
| PSI 05 | Retained surgical items | 14,186.64 | 5,726.63 | 2.477 | 0.013 | * |
| PSI 06 | Iatrogenic pneumothorax | 14,217.42 | 2,850.87 | 4.987 | < .001 | *** |
| PSI 07 | CVC bloodstream infection | 56,178.95 | 2,156.36 | 26.053 | < .001 | *** |
| PSI 08 | Fall with hip fracture | 29,632.88 | 4,759.48 | 6.226 | < .001 | *** |
| PSI 09 | Perioperative hemorrhage/hematoma | 15,422.67 | 1,019.65 | 15.125 | < .001 | *** |
| PSI 10 | Postoperative acute kidney injury | 95,061.65 | 3,511.04 | 27.075 | < .001 | *** |
| PSI 11 | Postoperative respiratory failure | 47,239.14 | 5,358.20 | 8.816 | < .001 | *** |
| PSI 12 | Perioperative embolism or thrombosis | 30,356.67 | 1,390.51 | 21.831 | < .001 | *** |
| PSI 13 | Postoperative sepsis | 58,504.80 | 2,393.97 | 24.438 | < .001 | *** |
| PSI 14 | Wound dehiscence | 40,760.01 | 4,070.07 | 10.015 | < .001 | *** |
| Age | | -136.77 | 16.23 | -8.426 | < .001 | *** |
| Sex: Male | | 5,310.00 | 613.55 | 8.655 | < .001 | *** |
| Nationality: Swiss | | -1,376.59 | 694.01 | -1.984 | 0.047 | * |
| Admission: Emergency | | 6,820.51 | 602.86 | 11.314 | < .001 | *** |
| Admission: From nursing home | | -4,280.90 | 2,397.22 | -1.786 | 0.074 |  |
| Admission: Transferred | | 22,709.20 | 1,189.27 | 19.095 | < .001 | *** |
| Elixhauser Index | | 1,027.72 | 22.86 | 44.966 | < .001 | *** |
| *Note*. *** = *p* < .001; ** = *p* < 0.01; * = *p* < 0.05; *B* unstandardized beta coefficients (costs), *t* test statistic, *p* probability value, *CHF* Swiss francs. For the estimation of the total national costs, the covariate "premature death" and adverse events from the obstetrical PSIs 17–PSI 19 were not included (see Section 2.3.3). | | | | | | |

Table S20 Sample characteristics of the complete national PSI data set and estimation of the total national costs due to adverse events covered by the PSIs in Switzerland in 2019.

|  |  | **Raw data** | | **Incidence** | **Costs (in CHF)** | **Sum (in CHF)** |
| --- | --- | --- | --- | --- | --- | --- |
|  | | *N*  (PSI = 0) | *N*  (PSI = 1) | Per thousand |  |  |
| PSI 02 | Death in low-mortality DRGs | 390,479 | 62 | 0.16 | -9,015.84 | -558,982.08 |
| PSI 03 | Pressure ulcer | 355,488 | 1,173 | 3.29 | 21,037.08 | 24,676,494.84 |
| PSI 04 | Death after serious complications | 6,511 | 1,333 | 186.51 | 43,118.33 | 57,476,733.89 |
| PSI 05 | Retained surgical items | 978,794 | 111 | 0.11 | 14,186.64 | 1,574,717.04 |
| PSI 06 | Iatrogenic pneumothorax | 929,319 | 434 | 0.47 | 14,217.42 | 6,170,360.28 |
| PSI 07 | CVC bloodstream infection | 679,179 | 748 | 1.10 | 56,178.95 | 42,021,854.60 |
| PSI 08 | Fall with hip fracture | 405,592 | 139 | 0.34 | 29,632.88 | 4,118,970.32 |
| PSI 09 | Postoperative hemorrhage/hematoma | 469,713 | 3,833 | 8.09 | 15,422.67 | 59,115,094.11 |
| PSI 10 | Postoperative acute kidney injury | 366,428 | 276 | 0.75 | 95,061.65 | 26,237,015.40 |
| PSI 11 | Postoperative respiratory failure | 354,828 | 339 | 0.95 | 47,239.14 | 16,014,068.46 |
| PSI 12 | Perioperative embolism or thrombosis | 491,524 | 1,823 | 3.70 | 30,356.67 | 55,340,209.41 |
| PSI 13 | Postoperative sepsis | 368,121 | 701 | 1.90 | 58,504.80 | 41,011,864.80 |
| PSI 14 | Wound dehiscence | 62,036 | 229 | 3.68 | 40,760.01 | 9,334,042.29 |
| PSI 15 | Accidental punctures or lacerations | 73,957 | 26 | 0.35 |  |  |
| PSI 17 | Birth trauma | 81,343 | 325 | 3.98 | 5,819.57 | 1,891,360.25 |
| PSI 18 | Obstetric trauma with instrument | 8,694 | 668 | 71.35 | 1,211.15 | 809,048.20 |
| PSI 19 | Obstetric trauma without instrument | 47,146 | 975 | 20.26 | 1,440.25 | 1,404,243.75 |
| **Sum (Switzerland)** | |  |  |  |  | **346,637,095.56** |

*Note*. The full data set (see Section 2.3.3) was used here to include patients with private supplementary insurance coverage as well. The total national costs were calculated by multiplying the number of PSI-related adverse events by their costs per case (extracted from Supplementary File 1, Tables S15–S17 and S19). PSI 15 had an insufficient number of cases to be used for cost analysis but is depicted here as well for the sake of completeness.

Table S21 POA-adjusted total national costs due to adverse events covered by the PSIs in Switzerland in 2019.

|  |  | **Raw data** | **POA** | **Adj. Nr. PSIs** | **Costs (in CHF)** | **Sum (in CHF)** |
| --- | --- | --- | --- | --- | --- | --- |
|  | | N (PSI = 1) | (%) | N (PSI = 1) |  |  |
| PSI 02 | Death in low-mortality DRGs | 62 | 0% | 62 | -9'015.84 | -558'982.08 |
| PSI 03 | Pressure ulcer | 1173 | 59% | 481 | 21'037.08 | 10'117'362.88 |
| PSI 04 | Death after serious complications | 1333 | 0% | 1333 | 43'118.33 | 57'476'733.89 |
| PSI 05 | Retained surgical items | 111 | 30% | 78 | 14'186.64 | 1'102'301.93 |
| PSI 06 | Iatrogenic pneumothorax | 434 | 8% | 399 | 14'217.42 | 5'676'731.46 |
| PSI 07 | CVC bloodstream infection | 748 | 19% | 606 | 56'178.95 | 34'037'702.23 |
| PSI 08 | Fall with hip fracture | 139 | 52% | 67 | 29'632.88 | 1'977'105.75 |
| PSI 09 | Postoperative hemorrhage/hematoma | 3833 | 8% | 3526 | 15'422.67 | 54'385'886.58 |
| PSI 10 | Postoperative acute kidney injury | 276 | 18% | 226 | 95'061.65 | 21'514'352.63 |
| PSI 11 | Postoperative respiratory failure | 339 | 0% | 339 | 47'239.14 | 16'014'068.46 |
| PSI 12 | Perioperative embolism or thrombosis | 1823 | 14% | 1568 | 30'356.67 | 47'592'580.09 |
| PSI 13 | Postoperative sepsis | 701 | 14% | 603 | 58'504.80 | 35'270'203.73 |
| PSI 14 | Wound dehiscence | 229 | 0% | 229 | 40'760.01 | 9'334'042.29 |
| PSI 15 | Accidental punctures or lacerations | 26 | 5% | 25 |  |  |
| PSI 17 | Birth trauma | 325 | 0% | 325 | 5'819.57 | 1'891'360.25 |
| PSI 18 | Obstetric trauma with instrument | 668 | 0% | 668 | 1'211.15 | 809'048.20 |
| PSI 19 | Obstetric trauma without instrument | 975 | 0% | 975 | 1'440.25 | 1'404'243.75 |
| **Sum (Switzerland)** | |  |  |  |  | **298'044'742.04** |

*Note*. The full data set (see Section 2.3.3) was used here to include patients with private supplementary insurance coverage as well. The total national costs were calculated by multiplying the number of PSI-related adverse events by their costs per case (extracted from Supplementary File 1, Tables S15–S17 and S19). PSI 15 had an insufficient number of cases to be used for cost analysis but is depicted here as well for the sake of completeness.
