# Supplementary material for: Assessing the excess costs of the in-hospital adverse events covered by the AHRQ’s Patient Safety Indicators in Switzerland
Source: PLoS One. 2024 Feb 5;19(2):e0285285. doi: 10.1371/journal.pone.0285285 (PMC10843032; doi:10.1371/journal.pone.0285285)
Supplement: S2 Appendix — (DOCX) [file pone.0285285.s002.docx]

supplementary file 2

**Results of the 1:1 matching in contrast to the 1:3 matching reported in the manuscript:**

Table S1 Sample characteristics and matching results

|  |  | **Raw Data** | | | | **Incidence** | | **After matching** | | **Matching rate** | **AUC** | |
| --- | --- | --- | --- | --- | --- | --- | --- | --- | --- | --- | --- | --- |
|  | | | *N* (PSI=0) | | *N* (PSI=1) | | Per thousand | *N* (PSI=0) | *N* (PSI=1) |  |  |  |
| PSI 02 | Death in low-mortality DRGs | 210,113 | | 43 | | 0.2 | | 41 | 43 | 100% | 0.919 | |
| PSI 03 | Pressure ulcer | 207,566 | | 865 | | 4.17 | | 865 | 865 | 100% | 0.744 | |
| PSI 04 | Death after serious complications | 6,511 | | 1,333 | | 204.73 | | 1,328 | 1333 | 100% | 0.731 | |
| PSI 05 | Retained surgical item | 666,990 | | 76 | | 0.11 | | 76 | 76 | 100% | 0.612 | |
| PSI 06 | Iatrogenic pneumothorax | 557,683 | | 310 | | 0.56 | | 310 | 310 | 100% | 0.726 | |
| PSI 07 | CVC bloodstream infection | 467,237 | | 553 | | 1.18 | | 551 | 553 | 100% | 0.826 | |
| PSI 08 | Fall with hip fracture | 186,552 | | 111 | | 0.6 | | 110 | 111 | 100% | 0.925 | |
| PSI 09 | Postoperative hemorrhage/hematoma | 303,722 | | 2,717 | | 8.95 | | 2,717 | 2,717 | 100% | 0.634 | |
| PSI 10 | Postoperative acute kidney injury | 230,484 | | 227 | | 0.98 | | 217 | 227 | 100% | 0.956 | |
| PSI 11 | Postoperative respiratory failure | 197,181 | | 90 | | 0.46 | | 87 | 90 | 100% | 0.921 | |
| PSI 12 | Perioperative embolism or thrombosis | 316,278 | | 1,393 | | 4.4 | | 1,391 | 1,393 | 100% | 0.861 | |
| PSI 13 | Postoperative sepsis | 231,525 | | 493 | | 2.13 | | 484 | 493 | 100% | 0.906 | |
| PSI 14 | Wound dehiscence | 43,545 | | 151 | | 3.47 | | 151 | 151 | 100% | 0.792 | |
| PSI 15 | Accidental punctures or lacerations | 43,794 | | 15 | | - | | - | - | - | - | |
| PSI 17 | Birth trauma | 12,865 | | 101 | | 7.85 | | 77 | 101 | 100% | 0.706 | |
| PSI 18 | Obstetric trauma with instrument | 7,515 | | 560 | | 74.52 | | 560 | 560 | 100% | 0.530 | |
| PSI 19 | Obstetric trauma without instrument | 39,964 | | 830 | | 20.77 | | 830 | 830 | 100% | 0.546 | |
| *Note. AUC* area under the curve. The matching rate indicates the proportion of cases that met the common support assumption. PSI 15 had an insufficient number of cases to be used for analysis but is depicted here as well for the sake of completeness. | | | | | | | | | | | |  |

Table S2 Comparisons of means and relative frequencies across matching variables

|  |  | **Age**^a^ | | **Sex**^b^ | | **Nationality** | | **Emergency** | | **Nursing home** | | **Transferred** | | **Elixhauser Index** | |
| --- | --- | --- | --- | --- | --- | --- | --- | --- | --- | --- | --- | --- | --- | --- | --- |
|  | PSI | 0 | 1 | 0 | 1 | 0 | 1 | 0 | 1 | 0 | 1 | 0 | 1 | 0 | 1 |
| PSI 02:  Death in low-mortality DRGs | Mean/RF | 72.68 | 77.56 | 56.10% | 51.16% | 63.41% | 79.07% | 65.85% | 74.42% | 14.63% | 16.28% | 4.88% | 11.63% | 8.27 | 6.98 |
|  | *SD* | 21.88 | 19.71 | - | - | - | - | - | - | - | - | - | - | 11.12 | 11.40 |
|  | *test stat.* | -0.78 | | 0.21 | | 2.52 | | 0.74 | | 0.04 | | 1.25 | | -0.58 | |
|  | *p* | .436 | | .650 | | .112 | | .391 | | .835 | | .434 | | .560 | |
| PSI 03:  Pressure ulcer ^d^ | Mean/RF | 77.72 | 78.35 | 53.76% | 53.53% | 81.27% | 80.92% | 80.92% | 82.89% | - | - | - | - | 15.74 | 17.46 |
|  | *SD* | 13.34 | 13.26 | - | - | - | - | - | - | - | - | - | - | 15.02 | 15.45 |
|  | *test stat.* | -1.25 | | 0.01 | | 0.03 | | 41.54 | | - | - | - | - | -2.40 | |
|  | *p* | .213 | | .923 | | .854 | | <.001 *** | | - | - | - | - | .016 * | |
| PSI 04:  Death after serious compli-cations | Mean/RF | 72.75 | 73.08 | 64.61% | 64.97% | 79.59% | 81.85% | 76.36% | 76.52% | 4.67% | 4.58% | 13.70% | 16.88% | 25.17 | 27.07 |
|  | *SD* | 12.90 | 13.33 | - | - | - | - | - | - | - | - | - | - | 16.59 | 16.48 |
|  | *test stat.* | -1.13 | | 0.04 | | 2.17 | | 0.01 | | 0.01 | | 5.17 | | -2.80 | |
|  | *p* | .259 | | 0.847 | | .141 | | .921 | | .910 | | .023* | | .005 ** | |
| PSI 05:  Retained surgical items | Mean/RF | 59.74 | 56.51 | 40.79% | 40.79% | 71.05% | 72.37% | 27.63% | 28.95% | 3.95% | 1.32% | 2.63% | 2.63% | 4.42 | 4.61 |
|  | *SD* | 17.94 | 18.92 | - | - | - | - | - | - | - | - | - | - | 9.19 | 11.45 |
|  | *test stat.* | -0.94 | | 0.00 | | 0.03 | | 0.03 | | 1.03 | | 0.00 | | -0.15 | |
|  | *p* | .348 | | > .999 | | .857 | | .857 | | .311 | | > .999 | | .884 | |
| PSI 06:  Iatrogenic pneumo-thorax | Mean/RF | 67.94 | 67.13 | 50.00% | 49.68% | 80.65% | 83.55% | 49.03% | 49.68% | 2.26% | 1.61% | 11.29% | 12.58% | 12.46 | 13.84 |
|  | *SD* | 16.85 | 15.92 | - | - | - | - | - | - | - | - | - | - | 14.73 | 13.28 |
|  | *test stat.* | -0.98 | | 0.01 | | 0.89 | | 0.03 | | 0.34 | | 0.25 | | -2.25 | |
|  | *p* | .326 | | .936 | | .346 | | .872 | | .560 | | .620 | | .024 * | |
| PSI 07:  CVC bloodstream infection | Mean/RF | 65.30 | 65.40 | 67.33% | 71.07% | 77.68% | 78.48% | 64.43% | 70.71% | 3.09% | 2.17% | 20.51% | 21.34% | 13.38 | 15.44 |
|  | *SD* | 18.91 | 15.74 | - | - | - | - | - | - | - | - | - | - | 15.01 | 14.64 |
|  | *test stat.* | -0.87 | | 1.81 | | 0.10 | | 4.96 | | 0.90 | | 0.12 | | -2.94 | |
|  | *p* | .386 | | 0.179 | | .747 | | .026 | | .342 | | .735 | | .003 ** | |
| PSI 08:  Fall with hip fracture | Mean/RF | 76.55 | 79.32 | 61.82% | 49.55% | 78.18% | 85.59% | 75.45% | 78.38% | 10.91% | 14.41% | 23.64% | 18.02% | 11.81 | 13.25 |
|  | *SD* | 14.24 | 12.09 | - | - | - | - | - | - | - | - | - | - | 11.78 | 12.78 |
|  | *test stat.* | -1.37 | | 3.37 | | 2.04 | | 0.27 | | 0.61 | | 1.06 | | -1.09 | |
|  | *p* | .170 | | .067 | | .153 | | .610 | | .433 | | .304 | | .277 | |
| PSI 09: Post-operative hemorrhage/hematoma | Mean/RF | 60.01 | 59.63 | 53.70% | 56.86% | 78.40% | 77.22% | 25.76% | 27.02% | 1.10% | 0.96% | 5.15% | 5.26% | 6.09 | 6.87 |
|  | *SD* | 18.41 | 19.68 | - | - | - | - | - | - | - | - | - | - | 11.75 | 11.50 |
|  | *test stat.* | -0.11 | | 5.51 | | 1.09 | | 1.10 | | 0.29 | | 0.03 | | -4.63 | |
|  | *p* | .912 | | .019* | | .296 | | .295 | | .591 | | .855 | | < .001 *** | |
| PSI 10:  Post-operative acute kidney injury | Mean/RF | 68.55 | 69.07 | 62.67% | 66.52% | 72.81% | 71.81% | 2.76% | 2.64% | 0.00% | 0.00% | 6.91% | 10.13% | 28.36 | 33.03 |
|  | *SD* | 14.33 | 13.27 | - | - | - | - | - | - | - | - | - | - | 19.36 | 16.86 |
|  | *test stat.* | -0.45 | | 0.72 | | 0.06 | | 0.01 | | - | | 1.09 | | -2.10 | |
|  | *p* | .650 | | .397 | | .813 | | .937 | | - | | .297 | | .036 * | |
| PSI 11:  Post-operative respiratory failure | Mean/RF | 68.56 | 67.17 | 72.41% | 83.33% | 74.71% | 78.89% | 0.00% | 2.22% | 0.00% | 1.11% | 6.90% | 6.67% | 21.68 | 26.77 |
|  | *SD* | 15.17 | 11.44 | - | - | - | - | - | - | - | - | - | - | 19.05 | 19.59 |
|  | *test stat.* | -1.46 | | 3.07 | | 0.43 | | 1.96 | | 0.97 | | 0.00 | | -1.55 | |
|  | *p* | .145 | | .080 | | .510 | | .497 | | .244 | | .952 | | .121 | |
| PSI 12:  Peri-operative embolism or thrombosis | Mean/RF | 68.07 | 69.15 | 53.20% | 53.41% | 81.09% | 82.91% | 49.32% | 54.70% | 2.59% | 3.16% | 12.65% | 13.14% | 16.63 | 19.53 |
|  | *SD* | 16.87 | 15.39 | - | - | - | - | - | - | - | - | - | - | 17.39 | 16.59 |
|  | *test stat.* | -0.97 | | 0.01 | | 1.57 | | 8.09 | | 0.81 | | 0.15 | | -5.77 | |
|  | *p* | 0.331 | | .911 | | .211 | | .004** | | .368 | | .703 | | < .001 *** | |
| PSI 13:  Post-operative sepsis | Mean/RF | 68.51 | 70.26 | 63.84% | 66.33% | 77.48% | 79.51% | 1.65% | 1.62% | 1.45% | 1.22% | 7.23% | 7.30% | 20.73 | 24.70 |
|  | *SD* | 15.91 | 13.21 | - | - | - | - | - | - | - | - | - | - | 18.10 | 17.52 |
|  | *test stat.* | -1.17 | | 0.66 | | 0.60 | | 0.00 | | 0.10 | | 0.00 | | -3.41 | |
|  | *p* | .242 | | .415 | | .439 | | .970 | | .755 | | .966 | | .001 ** | |
| PSI 14:  Wound dehiscence | Mean/RF | 69.44 | 69.77 | 56.95% | 66.23% | 78.81% | 82.78% | 47.68% | 49.01% | 1.32% | 0.66% | 7.95% | 8.61% | 14.34 | 14.82 |
|  | *SD* | 16.40 | 13.59 | - | - | - | - | - | - | - | - | - | - | 14.93 | 13.24 |
|  | *test stat.* | -10.83 | | 2.74 | | 0.77 | | 0.05 | | 0.34 | | 0.04 | | -0.39 | |
|  | *p* | <.001 *** | | .098 | | .381 | | .818 | | > .999 | | .835 | | .696 | |
| PSI 18:  Obstetric trauma with instrument ^e^ | Mean/RF | 33.38 | 33.12 | - | - | 54.46% | 55.54% | 56.25% | 56.43% | - | - | 1.07% | 0.71% | -0.38 | -0.59 |
|  | *SD* | 4.92 | 4.75 | - | - | - | - | - | - | - | - | - | - | 3.15 | 2.91 |
|  | *test stat.* | -0.90 | | - | | 0.13 | | 0.00 | | - | | 0.40 | | -1.50 | |
|  | *p* | .371 | | - | | .719 | | .952 | | - | | .525 | | .133 | |
| PSI 19:  Obstetric trauma without instrument ^e^ | Mean/RF | 32.67 | 32.64 | - | - | 61.45% | 59.04% | 54.46% | 56.14% | - | - | 0.12% | 0.36% | -0.51 | -0.57 |
|  | *SD* | 4.69 | 4.77 | - | - | - | - | - | - | - | - | - | - | 2.82 | 2.73 |
|  | *test stat.* | -0.21 | | - | | 1.01 | | 0.48 | | - | | 1.00 | | -0.11 | |
|  | *p* | .833 | | - | | .316 | | .489 | | - | | .625 | | .911 | |
|  |  | **Age (G)**^c^ | | **Sex** | | **Nationality: Swiss** | | **Birth weight** | | **Head size** | | **No. prev. live births^f^** | | **Elixhauser Index** | |
|  |  | 0 | 1 | 0 | 1 | 0 | 1 | 0 | 1 | 0 | 1 | 0 | 1 | 0 | 1 |
| PSI 17:  Birth traum | Mean/RF | -0.00 | 0.00 | 53.25% | 67.33% | 54.55% | 53.47% | 3,449.81 | 3,432.56 | 34.30 | 34.33 | 0.39 | 0.34 | 0.26 | 0.38 |
|  | *SD* | 0.98 | 1.02 | - | - | - | - | 517.27 | 577.34 | 5.83 | 5.15 | 0.61 | 0.66 | 1.34 | 1.82 |
|  | *test stat.* | -0.20 | | 3.65 | | 0.02 | | -0.28 | | -0.75 | | -0.84 | | -0.34 | |
|  | *p* | .839 | | .056 | | .886 | | .780 | | .451 | | .402 | | .737 | |
| *Note*. *** = *p* < .001; ** = *p* < 0.01; * = *p* < 0.05. Binary variables are expressed as relative frequencies (RF) in percent (%). Continuous variables are expressed as means and standard deviations (SD).  ^a^ Rounded up to 5 years. ^b^ 1 = male. ^c^ Gestational age. Gestational age is measured in weeks, but it was standardized to facilitate the interpretation. ^d^ In PSI 3, patients were excluded from the samples if they were transferred from another hospital or were admitted from a nursing home. ^e^ The variables sex and admission from a nursing home were excluded in PSI 18 and 19 because of missing relevance. *p* probability value. ^f^ Number of all previous live births of the mother. | | | | | | | | | | | | | | | |

Table S3 Comparisons of means and relative frequencies across outcome variables

|  |  | **Total cost in CHF** | | **LOS** | | **Number of readmissions** | | **Mortality** | |
| --- | --- | --- | --- | --- | --- | --- | --- | --- | --- |
|  | PSI | 0 | 1 | 0 | 1 | 0 | 1 | 0 | 1 |
| PSI 02:  Death in low-mortality DRGs | Mean/RF | 10,103.29 | 16,211.84 | 5.41 | 5.81 | 0.07 | 0.02 | 0.00% | 100.00% |
|  | *SD* | 9,274.51 | 18,100.84 | 6.66 | 6.57 | 0.26 | 0.15 |  | - |
|  | *test statistic* | -0.92 | | -0.23 | | -1.07 | | 84.00 | |
|  | *p* | .359 | | .819 | | .286 | | < .001 *** | |
| PSI 03:  Pressure ulcer | Mean/RF | 23,596.97 | 51,792.02 | 13.07 | 23.62 | 0.09 | 0.07 | 6.94% | 16.99% |
|  | *SD* | 31,424.85 | 77,644.00 | 10.92 | 24.21 | 0.31 | 0.26 | - | - |
|  | *test statistic* | -14.07 | | -14.17 | | -0.50 | | 41.54 | |
|  | *p* | < .001 *** | | < .001 *** | | .619 | | < .001 *** | |
| PSI 04:  Death after serious compli-cations | Mean/RF | 90,929.44 | 103,948.48 | 28.08 | 18.45 | 0.08 | 0.03 | 0.00% | 100.00% |
|  | *SD* | 101,175.76 | 121,130.00 | 24.83 | 20.52 | 0.28 | 0.19 | - | - |
|  | *test statistic* | -2.55 | | -16.48 | | -4.88 | | 2661.00 | |
|  | *p* | .011 * | | < .001 *** | | < .001 *** | | < .001 | |
| PSI 05:  Retained surgical items | Mean/RF | 14,159.43 | 30,748.18 | 5.83 | 10.78 | 0.04 | 0.07 | 1.32% | 2.63% |
|  | *SD* | 19,585.80 | 47,458.73 | 7.58 | 18.54 | 0.20 | 0.25 | - | - |
|  | *test statistic* | -3.44 | | -3.14 | | -0.72 | | 0.34 | |
|  | *p* | .001 * | | .002 ** | | .469 | | .560 | |
| PSI 06:  Iatrogenic pneumo-thorax | Mean/RF | 17,450.86 | 43,259.37 | 7.91 | 12.71 | 0.05 | 0.05 | 5.48% | 9.35% |
|  | *SD* | 26,675.60 | 59,343.67 | 8.75 | 12.86 | 0.22 | 0.21 | - | - |
|  | *test statistic* | -10.35 | | -6.11 | | -0.19 | | 3.38 | |
|  | *p* | < .001 *** | | < .001 *** | | .851 | | .066 | |
| PSI 07:  CVC bloodstream infection | Mean/RF | 21,975.91 | 96,456.09 | 9.58 | 28.25 | 0.07 | 0.11 | 5.08% | 10.85% |
|  | *SD* | 28,551.27 | 120,722.84 | 9.75 | 24.59 | 0.29 | 0.32 | - | - |
|  | *test statistic* | -20.10 | | -19.42 | | -2.79 | | 12.52 | |
|  | *p* | < .001 *** | | < .001 *** | | .005 ** | | < .001*** | |
| PSI 08:  Fall with hip fracture | Mean/RF | 34,597.71 | 65,256.16 | 12.35 | 24.60 | 0.10 | 0.06 | 4.55% | 17.12% |
|  | *SD* | 41,064.17 | 54,699.80 | 13.69 | 20.12 | 0.36 | 0.24 | - | - |
|  | *test statistic* | -5.98 | | -6.09 | | -0.57 | | 9.02 | |
|  | *p* | < .001 *** | | < .001 *** | | .569 | | .003** | |
| PSI 09: Post-operative hemorrhage/hematoma | Mean/RF | 17,438.13 | 35,766.92 | 5.96 | 11.84 | 0.03 | 0.25 | 1.36% | 2.50% |
|  | *SD* | 23,929.17 | 48,453.42 | 8.62 | 14.41 | 0.19 | 0.48 | - | - |
|  | *test statistic* | -26.75 | | -25.58 | | -21.16 | | 9.33 | |
|  | *p* | < .001 *** | | < .001 *** | | < .001 *** | | .002** | |
| PSI 10:  Post-operative acute kidney injury | Mean/RF | 43,208.13 | 184,824.96 | 11.70 | 33.07 | 0.03 | 0.05 | 4.15% | 38.77% |
|  | *SD* | 68,334.41 | 190,533.89 | 14.48 | 31.70 | 0.18 | 0.22 | - | - |
|  | *test statistic* | -14.04 | | -10.50 | | -1.07 | | 77.88 | |
|  | *p* | < .001 *** | | < .001 *** | | .284 | | < .001*** | |
| PSI 11:  Post-operative respiratory failure | Mean/RF | 30,761.25 | 140,710.77 | 9.41 | 31.54 | 0.02 | 0.06 | 4.60% | 25.56% |
|  | *SD* | 49,089.90 | 83,918.77 | 11.56 | 18.65 | 0.15 | 0.27 | - | - |
|  | *test statistic* | -9.62 | | -8.99 | | -0.80 | | 15.03 | |
|  | *p* | < .001 *** | | < .001 *** | | .426 | | < .001*** | |
| PSI 12:  Peri-operative embolism or thrombosis | Mean/RF | 35,672.24 | 72,030.42 | 11.49 | 21.65 | 0.06 | 0.10 | 7.05% | 10.19% |
|  | *SD* | 56,429.19 | 112,568.59 | 14.38 | 22.59 | 0.26 | 0.31 | - | - |
|  | *test statistic* | -20.11 | | -19.36 | | -3.93 | | 8.76 | |
|  | *p* | < .001 *** | | < .001 *** | | < .001 *** | | .003** | |
| PSI 13:  Post-operative sepsis | Mean/RF | 30,759.35 | 127,730.62 | 8.88 | 32.46 | 0.04 | 0.10 | 2.27% | 23.33% |
|  | *SD* | 54,551.51 | 147,951.07 | 12.14 | 27.79 | 0.21 | 0.30 | - | - |
|  | *test statistic* | -20.86 | | -20.54 | | -3.78 | | 96.37 | |
|  | *p* | < .001 *** | | < .001 *** | | < .001 *** | | < .001*** | |
| PSI 14:  Wound dehiscence | Mean/RF | 33,367.01 | 78,382.95 | 12.18 | 26.76 | 0.06 | 0.17 | 5.30% | 10.60% |
|  | *SD* | 40,476.75 | 63,757.46 | 14.67 | 14.48 | 0.24 | 0.40 | - | - |
|  | *test statistic* | -10.83 | | -10.67 | | -2.92 | | 2.90 | |
|  | *p* | < .001 *** | | < .001 *** | | .004 ** | | .089 | |
| PSI 17:  Birth trauma | Mean/RF | 5,352.52 | 14,611.43 | 4.65 | 6.93 | 0.04 | 0.02 | - | - |
|  | *SD* | 9,169.26 | 20,723.45 | 3.34 | 6.35 | 0.19 | 0.14 | - | - |
|  | *test statistic* | -5.53 | | -2.63 | | -0.76 | | - | |
|  | *p* | < .001 *** | | .009 ** | | .445 | | - | |
| PSI 18:  Obstetric trauma with instrument | Mean/RF | 8,203.21 | 9,400.23 | 4.03 | 4.30 | 0.00 | 0.00 | - | - |
|  | *SD* | 4,178.72 | 3,445.03 | 1.35 | 1.43 | 0.00 | 0.00 | - | - |
|  | *test statistic* | -7.63 | | -3.61 | | 0.00 | | - | |
|  | *p* | < .001 *** | | < .001 *** | | - | | - | |
| PSI 19:  Obstetric trauma without instrument | Mean/RF | 6,618.26 | 7,851.62 | 3.45 | 3.83 | 0.00 | 0.00 | - | - |
|  | *SD* | 2,622.01 | 2,689.39 | 1.22 | 1.18 | 0.02 | 0.00 | - | - |
|  | *test statistic* | -10.43 | | -7.21 | | -1.00 | | - | |
|  | *p* | < .001 *** | | < .001 *** | | .317 | | - | |
| *Note*. *** = *p* < .001; ** = *p* < 0.01; * = *p* < 0.05; Binary variables are expressed as relative frequencies (RF) in percent (%). Continuous variables are expressed as means and standard deviations (SD). *CHF* Swiss francs, *LOS* length of stay, *p* probability value. | | | | | | | | | |

Table S4 Overview of the excess costs of PSI-related adverse events from the individual regression analyses using 1:1 matching

|  | | ***B (in CHF)*** | ***Std. Error*** | ***t*** | ***p*** |  |
| --- | --- | --- | --- | --- | --- | --- |
| PSI 02 | Death in low-mortality DRGs | 8,271.76 | 3,238.43 | 2.554 | .013 | ***** |
| PSI 03 | Pressure ulcer | 27,419.96 | 2,730.19 | 10.043 | < .001 | ******* |
| PSI 04 | Death after serious complications | 23,767.24 | 4,208.20 | 5.648 | < .001 | ******* |
| PSI 05 | Retained surgical item | 15,424.33 | 5,303.46 | 2.908 | < .001 | ******* |
| PSI 06 | Iatrogenic pneumothorax | 25,502.31 | 3,637.86 | 7.010 | < .001 | ******* |
| PSI 07 | CVC bloodstream infection | 69,763.31 | 5,000.20 | 13.952 | < .001 | ******* |
| PSI 08 | Fall with hip fracture | 29,743.94 | 5,941.70 | 5.006 | < .001 | ******* |
| PSI 09 | Perioperative hemorrhage/hematoma | 17,284.63 | 940.38 | 18.381 | < .001 | ******* |
| PSI 10 | Postoperative acute kidney injury | 137,984.64 | 13,340.60 | 10.343 | < .001 | ******* |
| PSI 11 | Postoperative respiratory failure | 104,678.38 | 10,055.08 | 10.410 | < .001 | ******* |
| PSI 12 | Perioperative embolism or thrombosis | 32,226.37 | 3,173.08 | 10.156 | < .001 | ******* |
| PSI 13 | Postoperative sepsis | 92,699.76 | 6,844.87 | 13.543 | < .001 | ******* |
| PSI 14 | Wound dehiscence | 44,126.61 | 5,810.23 | 7.595 | < .001 | ******* |
| PSI 17 | Birth trauma | 7,664.09 | 2,384.71 | 3.214 | .002 | ****** |
| PSI 18 | Obstetric trauma with instrument | 1,201.98 | 22 8.70 | 5.256 | < .001 | ******* |
| PSI 19 | Obstetric trauma without instrument | 1,245.88 | 130.01 | 9.583 | < .001 | ******* |
| *Note*. *** = *p* < .001; ** = *p* < 0.01; *B* unstandardized beta coefficients (costs), *t* test statistic, *p* probability value, *CHF* Swiss francs. | | | | | | |
